# Population genomics of fall armyworm by genotyping-by-sequencing: Implications for pest management

**DOI:** 10.1371/journal.pone.0284587

**Published:** 2023-04-18

**Authors:** Tamylin Kaori Ishizuka, Erick Mauricio Goes Cordeiro, Alessandro Alves-Pereira, Carlos Eduardo de Araújo Batista, María Gabriela Murúa, José Baldin Pinheiro, Amit Sethi, Rodney N. Nagoshi, Josemar Foresti, Maria Imaculada Zucchi

**Affiliations:** 1 Institute of Biology, State University of Campinas, Campinas, Brazil; 2 Corteva Agriscience, Mogi Mirim, São Paulo, Brazil; 3 Department of Entomology, University of São Paulo–ESALQ/USP, Piracicaba, Brazil; 4 Department of Genetics, University of São Paulo–ESALQ/USP, Piracicaba, Brazil; 5 Instituto de Bioprospección y Fisiología Vegetal (INBIOFIV), CONICET-UNT, Tucumán, Argentina; 6 Corteva Agriscience, Johnston, Iowa, United States of America; 7 United States Department of Agriculture-Agricultural Research Service, Gainesville, Florida, United States of America; 8 Agência Paulista de Tecnologia dos Agronegócios, Pólo Regional Centro-Sul, Piracicaba, Brazil; ICAR Research Complex for North Eastern Hill Region Manipur Centre, INDIA

## Abstract

The fall armyworm (FAW), *Spodoptera frugiperda*, is a significant pest of many crops in the world and it is native to the Americas, where the species has shown the ability to rapidly evolve resistance to insecticides and transgenic plants. Despite the importance of this species, there is a gap in the knowledge regarding the genetic structure of FAW in South America. Here, we examined the genetic diversity of FAW populations across a wide agricultural area of Brazil and Argentina using a Genotyping-by-Sequencing (GBS) approach. We also characterized samples by their host strain based on mitochondrial and Z-linked genetic markers. The GBS methodology enabled us to discover 3309 SNPs, including neutral and outlier markers. Data showed significant genetic structure between Brazil and Argentina populations, and also among the Argentinian ecoregions. Populations inside Brazil showed little genetic differentiation indicating high gene flow among locations and confirming that structure is related to the presence of corn and rice strains. Outlier analysis indicated 456 loci putatively under selection, including genes possibly related to resistance evolution. This study provides clarification of the population genetic structure of FAW in South America and highlights the importance of genomic research to understand the risks of spread of resistance genes.

## Introduction

The fall armyworm (FAW), *Spodoptera frugiperda* (J. E. Smith) (Lepidoptera: Noctuidae), is a major agricultural pest that can feed on several different hosts [1]. FAW have the ability to evolve rapid resistance to insecticides and transgenic crops, which can impact the effectiveness of control strategies [2]. The spread of such resistance traits is dependent on the migratory behavior of FAW and it is therefore important for effective pest management to delineate the pattern and magnitude of population movement across national boundaries.

In North America, FAW populations from Texas and Florida make annual migrations northwards to recolonize areas in the north of USA and Canada [3]. This behavior reflects the inability of FAW to survive freezing temperatures, which limits winter populations to the most southern locations in the states of Texas and Florida [4]. Less is known about migration patterns in South America, which experiences a significantly different climate than North America. Climate suitability modeling shows that South America features suitable conditions for persistent FAW populations in this continent, except for most of Argentina [5]. This suggests an annual migration towards Argentina from more suitable locations, with the neighboring countries being the prime candidates for the migratory sources [6].

A complicating factor for FAW is that the species can be subdivided into two groups called host strains that differ in their distribution on plant hosts in the field, with the C-strain preferentially found in corn and the R-strain in pasture grasses and, to a lesser extent, rice [7, 8]. The host strains are morphologically indistinguishable and so can only be identified through the use of molecular markers, with the most commonly used being polymorphisms in the mitochondrial cytochrome oxidase subunit I (COI) and the Z-chromosome linked triosephosphate isomerase (Tpi) genes [9, 10].

At this time, the most consistent evidence of population structure in South America is that indicative of the two host strains, although some level of genetic structure has been observed for populations within Paraguay and within Brazil [11–13]. Overall, it remains unclear whether and to what extent geographically separated FAW in South America exhibit genetic differentiation. To better address this issue, we used Genotyping-by-Sequencing (GBS), a variation of the commonly used restriction-site-associated DNA sequencing methodology to identify single nucleotide polymorphisms (SNPs). GBS data allows us to resolve patterns of genetic diversity and spatial structure at very fine scale [14], and it was utilized in this study to evaluate patterns of gene flow and genetic structure of FAW populations across Brazil and Argentina. We additionally examined the information of host strains to discover informative loci putatively under selection. According to the results, we discuss some practical implications of our findings to the FAW management in South America.

## Material and methods

### Sampling and DNA extraction

**Fig 1:**
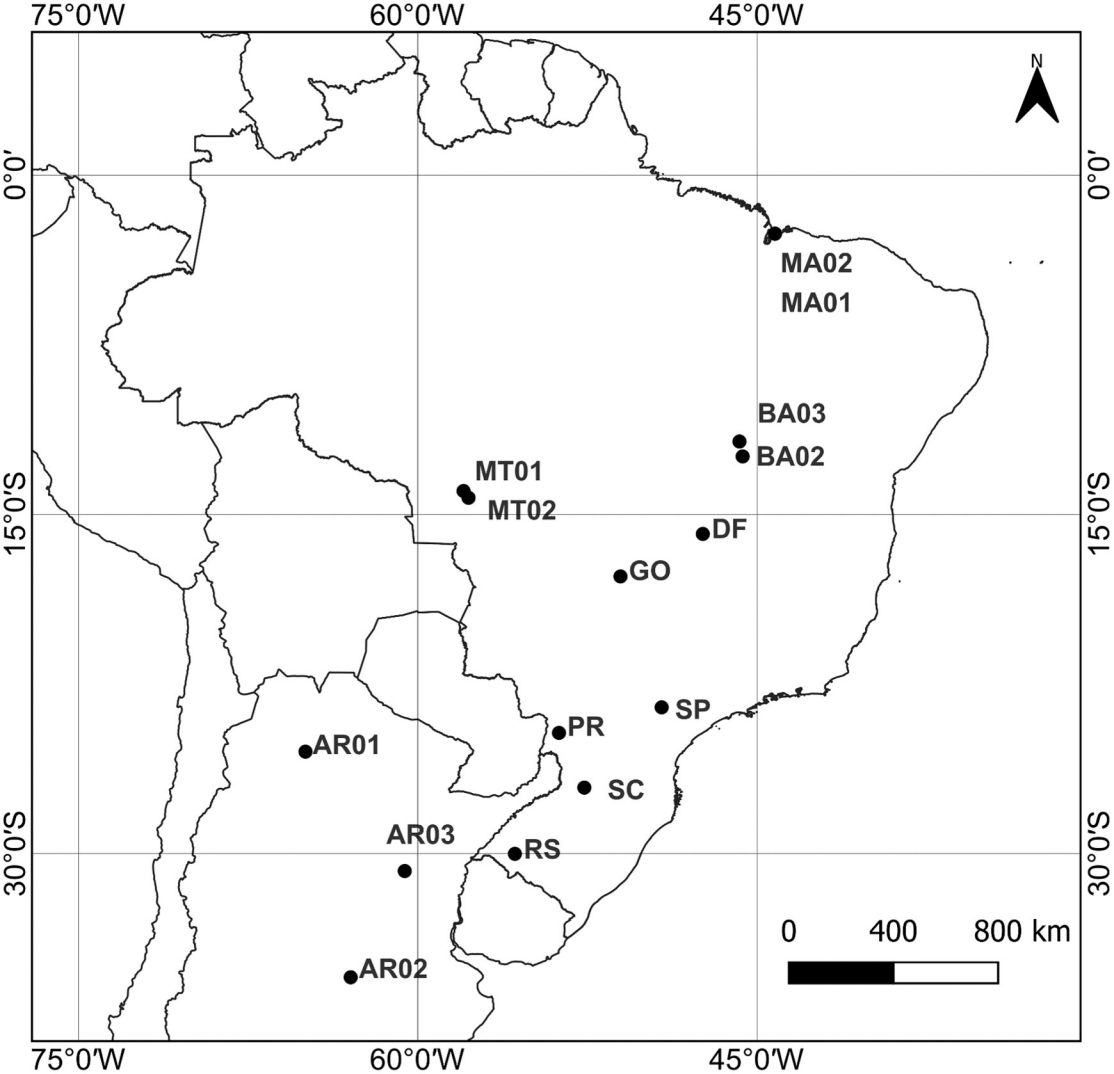
Fall armyworm sampling locations in Brazil and Argentina according to ecoregions. Shapefile of limits of countries in South America: IBGE-Mapas (IBGE—Brazilian Institute of Geography and Statistics; Available at: <https://geoftp.ibge.gov.br/cartas_e_mapas/bases_cartograficas_continuas/bc250/versao2021/shapefile/bc250_shapefile_2021_11_18.zip>; Acessed on February 26, 2023) QGIS v3.28.3 (Available at: <https://qgis.org/en/site/>; Acessed on: February 26, 2023). DATUM: SIRGAS 2000.

Fall armyworm caterpillars were manually collected in fields from 12 locations in Brazil under the SISBIO license number 58435, and from 3 locations of Argentina, between June of 2018 and January of 2021 (Fig 1, Table 1). Larvae were transferred to trays containing artificial diet and reared in laboratory conditions until become moths. Moths were placed in 1.5 mL polypropylene microcentrifuge tubes with 98% ethanol and stored at -20ºC. Species identification was confirmed by morphology and sequencing of the COI gene (see below). We extracted DNA from the legs of random adults using CTAB-based method [15]. DNA quality and quantification were assayed by agarose electrophoresis gel (1% w/v) stained with SYBR Safe DNA Gel Stain (Invitrogen, Carlsbad, CA, USA). DNA concentrations were adjusted to approximately 30 ng/μL.

**Table 1:** Fall armyworm (*Spodoptera frugiperda*) sampling information. N refers to the number of samples successfully sequenced after GBS processing, totaling 228 individuals.

Location Code	Country	Locality (City, State)	Collection Date	Latitude	Longitude	Host	N
AR01	Argentina	Metán—Salta	22-Feb-18	-25.5	-64.97	Corn	11
AR02	Argentina	America—Buenos Aires	24-Jan-18	-35.48	-62.97	Corn	3
AR03	Argentina	San Justo—Santa Fe	27-Feb-18	-30.78	-60.58	Corn	15
BA02	Brazil	São Desidério—Bahia	26-Jun-18	-12.44	-45.64	Corn	19
BA03	Brazil	Barreiras—Bahia	19-Jul-18	-11.78	-45.78	Corn	18
DF	Brazil	Planaltina—Distrito Federal	8-Jun-18	-15.87	-47.4	Corn	20
GO	Brazil	Rio Verde—Goiás	22-Jun-18	-17.75	-51.04	Corn	24
MA01	Brazil	São Luís—Maranhão	12-Dec-18	-2.59	-44.21	Corn	10
MA02	Brazil	São Luís—Maranhão	19-Dec-18	-2.58	-44.21	Corn	13
MT01	Brazil	Campo Novo do Parecis—Mato Grosso	25-Jun-18	-13.97	-57.98	Corn	24
MT02	Brazil	Campo Novo do Parecis—Mato Grosso	1-Jul-18	-14.27	-57.76	Corn	21
PR	Brazil	Toledo—Paraná	20-Jun-18	-24.67	-53.76	Corn	9
RS	Brazil	Alegrete—Rio Grande do Sul	13-Jan-21	-30.02	-55.71	Rice	3
SC	Brazil	Chapecó—Santa Catarina	14-Dec-20	-27.09	-52.64	Corn	15
SP	Brazil	Taquarituba—São Paulo	14-Jul-18	-23.54	-49.22	Corn	23

Distribution map of the populations was drawn using the software QGIS v3.28.3-Firenze. (Open Access Geographic Information System, https://qgis.org/en/site/. Accessed on February 26^th^, 2023). Publicly available shapefile of South American country boundaries was downloaded from IBGE-Mapas (IBGE–Brazilian Institute of Geography and Statistics, https://geoftp.ibge.gov.br/cartas_e_mapas/bases_cartograficas_continuas/bc250/versao2021/shapefile/bc250_shapefile_2021_11_18.zip. Accessed on February 26^th^, 2023).

### Host strains identification

For the 228 specimens whose reads were successfully retained after GBS processing, we did polymerase chain reaction (PCR) amplifications of the COI and TpiEI4 regions to characterize host strains based on diagnostic polymorphisms at mCOI1164, mCOI1287 and gTpi183 positions using the same primer sequences and procedures as described elsewhere [4]. Samples featuring both C and T nucleotides at the gTpi183 position were identified as hybrids (*Tpi*H). The COI sequences were used to confirm species identification as well. Sequences were aligned with Clustal W [16] using MEGA 7 [17]. The genetic data presented in this study are publicly available on GenBank (BioProject PRJNA847933, and accession numbers ON704174—ON704629).

### GBS library sequencing and data processing

The steps of the GBS library preparation were done according to methodology described elsewhere [18] with the following modifications. Genomic DNA was digested with restriction enzymes *Nsi*I-*Mse*I (New England Biolabs—NEB, Ipswich, MA, USA). The barcoded *Nsi*I adapters and a common *Mse*I adapter were ligated to the digested DNA of each sample. Barcoded DNA fragments from all samples were pooled in a single tube and amplified by PCR. The libraries were single-end sequenced to 150 nucleotides on a single lane using the Illumina NextSeq500/550 sequencing kit v2 (Illumina, Inc. San Diego, CA, USA) at the Genome Investigation and Analysis Laboratory of University of São Paulo.

Sequencing quality of GBS libraries was evaluated using FastQC [19]. The 3’end of raw reads were trimmed to 90 bp and were inspected for adaptor sequences removal. We performed demultiplex using *process-radtags* in STACKS v.1.42 [20]. Reads could be assigned to each individual based on the sequence of the barcodes. Samples with less than 200,000 sequences and/or unexpected GC contents were removed for further analysis. Reads were aligned to the *Spodoptera frugiperda* genome *ZJU_Sfru_1*.*0*, under Bioproject PRJNA590312 [21], using the algorithm BWA-MEM of the software BWA 0.7.17 [22]. Alignment files were processed with SAMtools [23] and Picard (http://broadinstitute.github.io/picard). We identified SNPs using freebayes 1.3.4 [24] with --standard_filters option. Filtering was performed using VCFtools v0.1.12a [25] and BCFtools 0.1.12 [22]. We retained bi-allelic SNPs that passed the following criteria: minor allele frequency ≥ 0.01, read depth ≥ 5X, mapping quality ≥ 20, maximal amount of missing data per locus = 10%. Variants were separated by a minimum distance of 150 bp and r^2^ threshold of 0.6. Results were stored in variant call format (VCF) after an additional filter to remove six samples with more than 25% of missing data, and SNPs identified at sexual chromosome W present only in females, mitochondrial genome or in unanchored contigs of the reference genome.

### Genetic differentiation analysis

Genetic diversity was estimated by calculating the observed heterozygosity H_O_, expected heterozygosity H_E_, nucleotide diversity π, the inbreeding coefficients (F_IS_), and the pairwise Fixation Index (F_ST_) using *hierfstat* package [26, 27]. We tested for significant differences in heterozygosities and F_IS_ using confidence intervals calculated based on 1000 bootstrap resamples. F_ST_ relations were illustrated by heatmaps generated by the *RColorBrewe*r R package. Genetic structure was additionally explored through the principal component analysis (PCA) and the discriminant analysis of principal components (DAPC) using *ade4* [28] and *adegenet* [29, 30] for R software [31]. Specimens were grouped by the ADMIXTURE v1.3.0 software, and the best value of inferred genetic groups (K) was implemented by the cross-validation method [32].

### Outlier SNPs detection and annotation

We searched for loci putatively under selection in the set of 15 populations and in the set of strains, where samples were identified as R- or C- strains using the *Tpi* marker as previously described [4]. *Tpi*H individuals were not included in this analysis. Outlier SNPs were identified using three methods. The *fsthet* package [33] generates smoothed quantiles for the F_ST_–heterozygosity relationship from the empirical distribution, and outliers were selected with a 95% confidence level threshold. The *pcadapt* package [34] considers population structure determined by PCA, and outliers were identified using a false discovery rate (FDR) of 0.05. The program FLK [35] assumes that SNPs were polymorphic in an ancestral population, and uses a population tree to build a null model of the behavior of F_ST_. We retained candidate markers identified by at least two methods and the remaining loci were considered as neutral. We compared the sequences containing outliers with the annotation files of the reference genome to identify loci present in encoding genes. We generated a fasta file containing the protein sequences to run Blast2GO [36] software using blastp with Insecta Taxa, InterPro Scan, GO mapping and functional annotation. The GO terms for each gene were submitted to Web Gene Ontology Annotation Plot (WEGO) for summarization [37].

## Results

### Fall armyworm host strains

**Fig 2:**
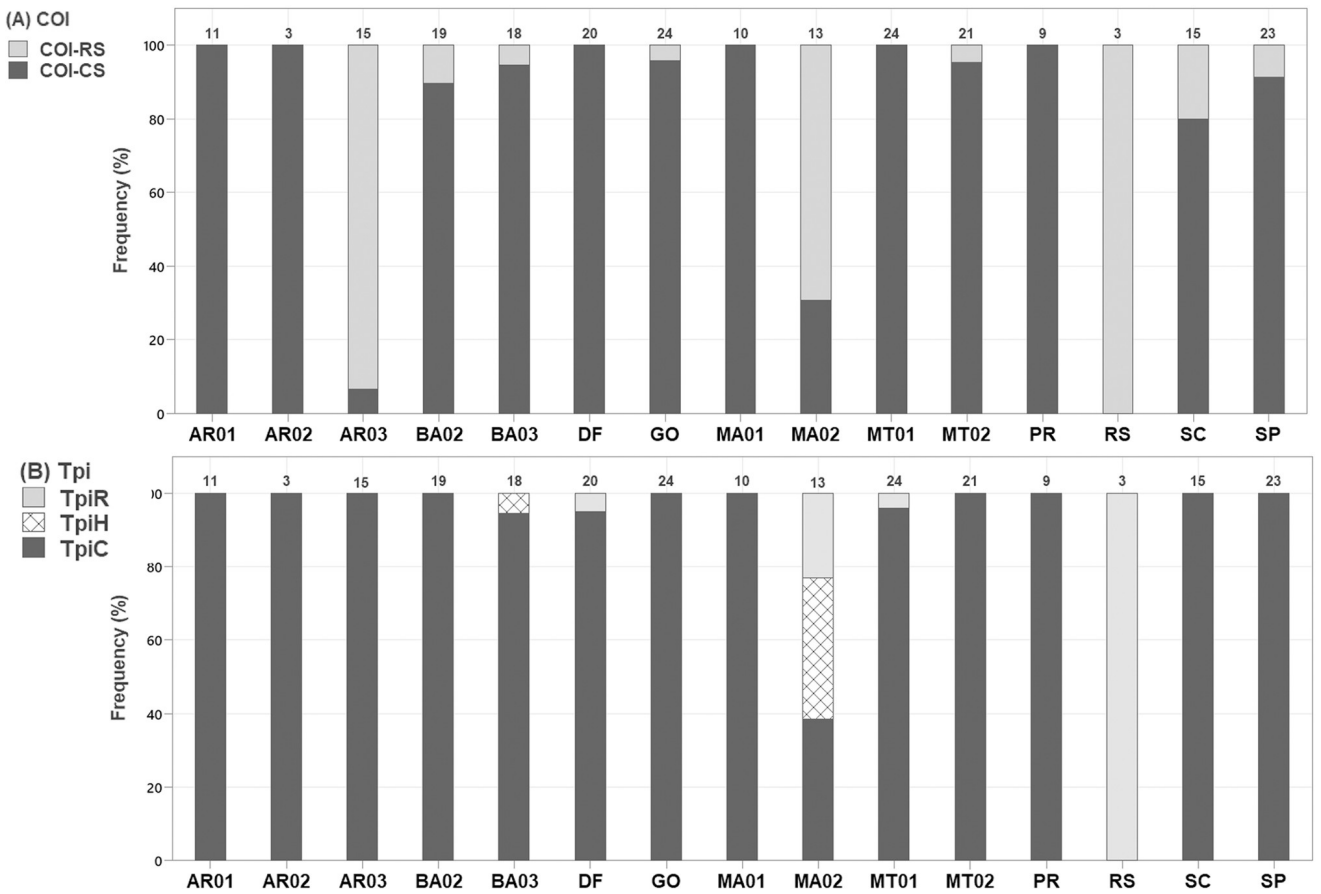
Haplotype frequencies in fall armyworm populations of Brazil and Argentina using COI and *Tpi* markers. A) COI strains COI-RS (R-strain) and COI-CS (C-strain) B) *Tpi* marker identifies R-strain (*Tpi*R), C-strain (*Tpi*C), and hybrids (*Tpi*H) that feature both diagnostic polymorphisms at site gTpi183.

Utilizing diagnostic polymorphisms in the COI and Tpi regions, we were able to determine the strain composition of the fall armyworm populations. A higher percentage of the samples collected from corn fields were identified as C-strain using the *Tpi* marker (95%) than COI (86%), consistent with other studies suggesting that *Tpi* is a better strain marker than COI [10]. The AR03 population in Argentina (Santa Fe) had the lowest agreement between markers (7%). *Tpi*-hybrids (*Tpi*H) were found only in Brazilian locations, mainly in MA02 population, which also had many R-strain samples (Fig 2).

### SNPs discovery

The GBS library generated a total of 187,842,557 reads that were retained after demultiplexing and quality checking. A total of 3309 SNP loci were retained in 228 individuals from 15 locations across Brazil and Argentina. Mean sequencing depth per SNP was 23.3 x (min = 8.7x, max = 45.1x). Estimates of genetic diversity were similar across locations and are summarized in S1 Table.

### Gene flow and population genetic structure

**Fig 3:**
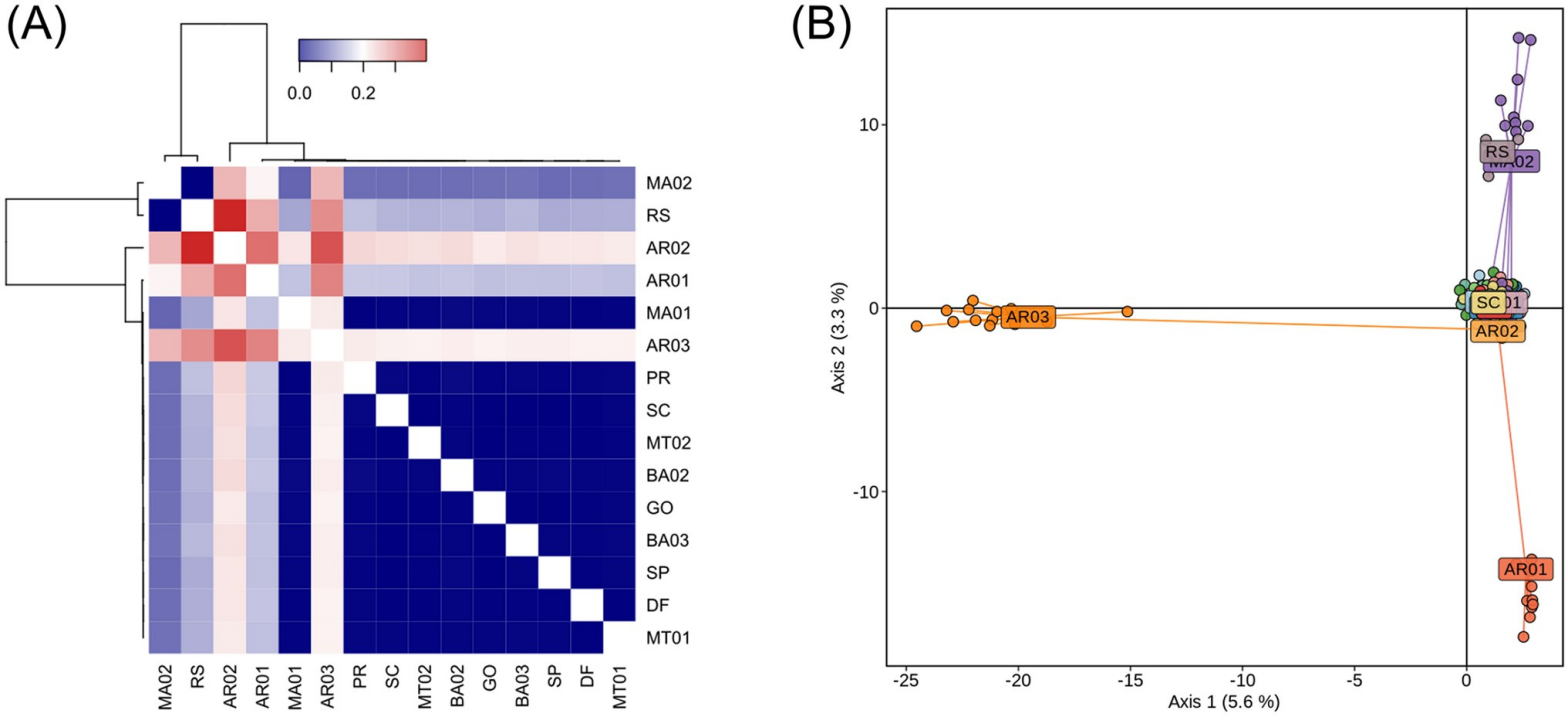
Genetic structure of FAW populations from Brazil and Argentina based on 3309 SNP loci. (A) Heatmap and dendrogram based on F_ST_ pairwise distances among 15 locations. Red color represents a greater degree of differentiation. (B) Principal Component Analysis (PCA) showing the first two components. Geographic locations are represented by different colors, and dots represent different individuals.

Pairwise F_ST_ calculations (S1 Fig, Fig 3A) and PCA analysis (Fig 3B) revealed high levels of genetic differentiation among the three populations from Argentina, ranging from 0.3742 to 0.4305, which is a strong indicative of reduced gene flow among these locations. On the other hand, Brazilian populations belonging to C-strain had very low F_ST_ values (ranging from 0.000 to 0.006). Likewise, Brazilian R-strain populations MA02 and RS had pairwise F_ST_ = 0. Lower pairwise F_ST_ distances (S3 Fig) can be a result of lower divergence time in addition to the presence of gene flow across Brazil. Aditionally, the two first components of PCA grouped Brazilian populations by their host strains, and showed that Argentinean population AR02 was more related to the Brazilian C-strain populations than to the Argentinean AR01 and AR03.

### Outlier detection

**Fig 4:**
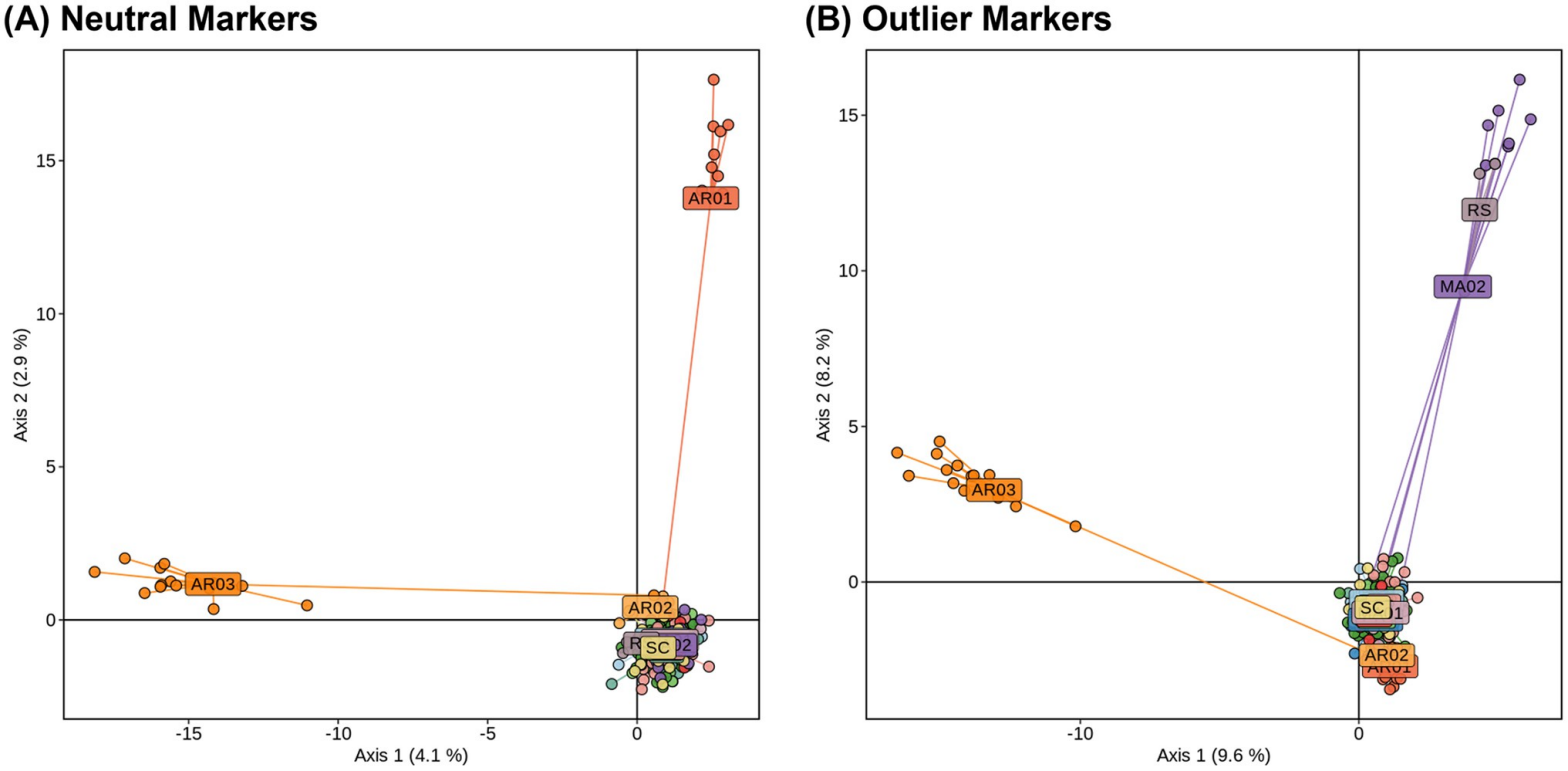
PCA analysis generated with (A) 2,853 neutral SNPs, (B) 456 outliers. Dots represent different individuals of each location.

**Fig 5:**
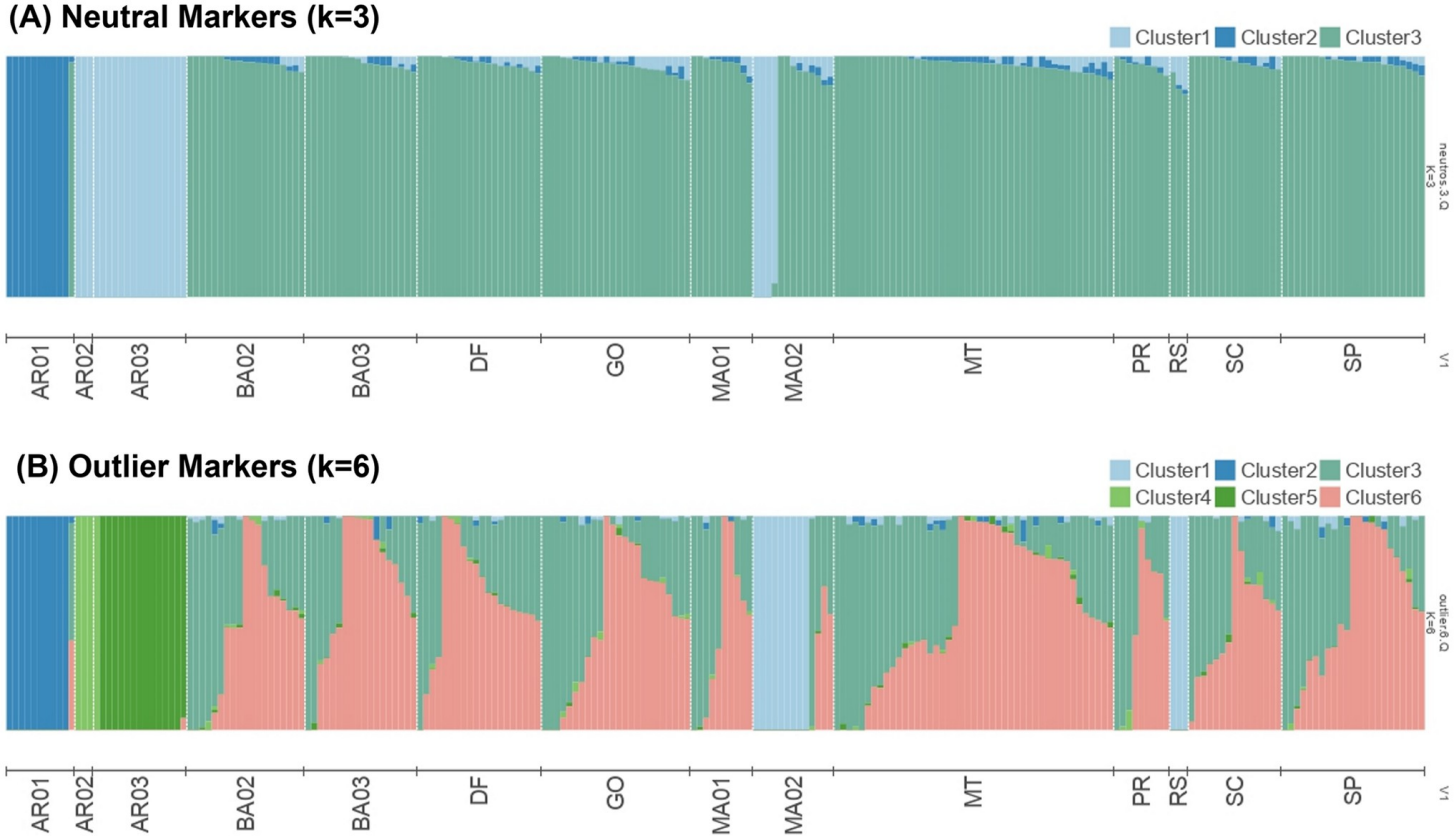
Admixture analysis of fall armyworm populations based on (A) 2,853 neutral SNP loci and (B) 456 outlier SNP loci. Bar plot colors indicate estimated proportions of ancestry for each individual shown as a vertical line.

When considering the 15 FAW populations, we identified 209 outlier SNPs in common by at least two of the three tests performed, whereas by comparing R- and C-strains we found 293 outliers in common by at least two of the three tests performed (S4 Fig). Altogether, we identified a total of 456 outlier loci putatively under selection (13.8%), and the remaining 2853 SNPs were considered to be neutral. By analyzing only neutral markers, the PCA plot indicated three clusters, grouping the AR02 (Buenos Aires) closest to the Brazilian populations (Fig 4A). Analysis of populations using Admixture showed that four individuals from MA02 featured similar admixture patterns as FAW individuals from AR02 and AR03 (Fig 5A). The outlier loci in turn resulted in four clusters: C-strain-Argentina, AR03, C-strain-Brazil, and R-strain-Brazil (Fig 4B). When considering loci under positive selection, an increased number of distinct genetic pools was obtained in Admixture analysis (K = 6), and more individuals appeared to feature non-admixed pattern (Fig 5B).

### Annotation of outlier loci

**Fig 6:**
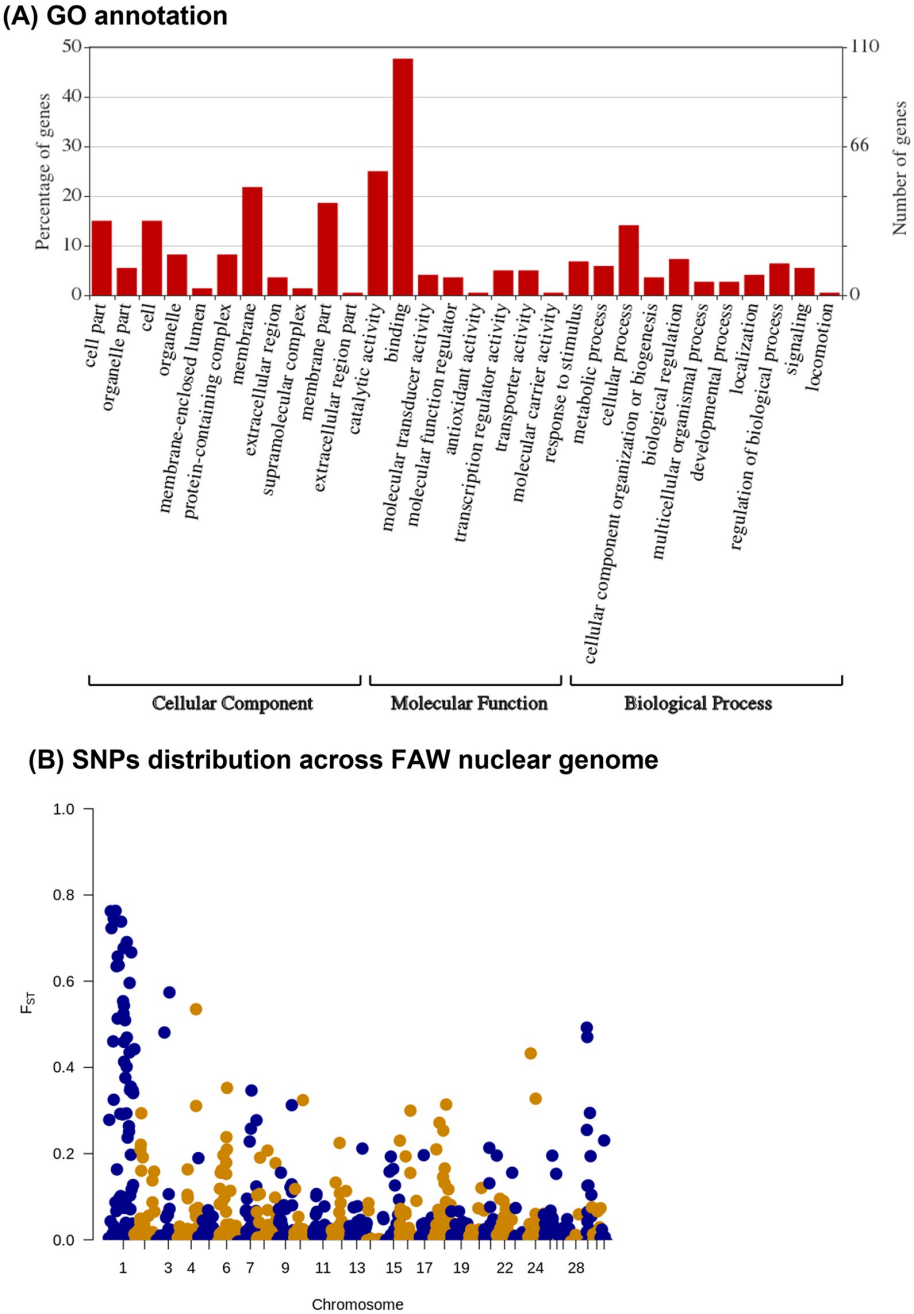
Outlier SNPs under positive selection using Pcadapt, FstHet, and FLK. (A) GO categories of putative loci under selection generated from WEGO. The results are presented in three main categories: biological process, cellular component, and molecular function. The left y-axis indicates the percentage of a specific category of genes in the main category. The right y-axis indicates the number of genes in each category. (B) Manhattan plot of F_ST_ analysis comparing C-strain and R-strain against the position of SNPs on each of the 31 chromosomes. Chromosome 1 corresponds to the sex chromosome Z. Each SNP is represented by a dot. Chromosomes are represented by blue (odd) and orange (even).

For outlier analyses, 456 SNP loci putatively under selection were compared to the annotations of the genome of a specimen collected in China (*ZJU_Sfru_1*.*0*) and 306 of these were within predicted protein coding genes of FAW. After Blast2GO analysis, 220 proteins were successfully mapped, and 94 were annotated (S2 Table). We found many outlier loci within genes possibly involved in binding functions and associated with the cell membrane (Fig 6A). Among the 94 loci with GO IDs, SNP_1055 was annotated as an ABC transporter C subfamily member 13, and the mutation was putatively under selection when comparing different locations, rather than related to the presence of two host strains. Regarding detoxifying activity, the locus SNP_3077 was in a gene similar to cytochrome P450 CYP314A1 (this locus was detected as outlier when comparing host strains), and the locus SNP_1559 was in a gene similar to an annotated esterase FE4-like. Another mechanism of insect defense involves chitin processing, and here we had five loci related to cuticle proteins or chitin binding (SNP_574, SNP_1209, SNP_1853, SNP_2799, SNP_3139).

Analysis of outliers comparing host strains resulted in 68 outlier SNPs (23% of 293) in the sex chromosome Z, which concentrated over twice the number of outliers compared to the autosomal chromosomes (Fig 6B). The locus SNP_421 located at chromosome 3 was within a gene similar to an odorant receptor and could be related to sensorial stimulus to either host perception or male mating preferences. The locus SNP_3298 was in a gene that had 100% identity to a GPI-anchored glycoprotein. Besides this locus, another 188 loci were successfully blasted with more than 90% similarity, but had no GO ID associated with them (S3 Table), including one locus identified as a cytochrome P450 (SNP_423), one cadherin (SNP_1010), one zinc carboxypeptidase (SNP_1352), one GABA receptor (SNP_719), and one UDP-glucosyltransferase (SNP_2719). The frequencies of each polymorphism for some candidate genes under selection were also calculated for the Brazil locations (S5 Fig).

## Discussion

GBS has been used successfully in insect pests to reveal insights about gene flow and coancestry [38], spatial and temporal genetic structure [39, 40], and incursions of invasive pests [41, 42]. Here we associated the informative data set provided by GBS with molecular markers used for host strain identification to better explain the patterns of FAW population structure in Brazil and Argentina and to identify candidate genes putatively under selection.

By assessing a large number of samples in Brazil, we confirmed that FAW collected in corn fields were predominantly C-strain, with less than 4% of samples featuring both the COI-RS and *Tpi*R diagnostic markers, and the few specimens featuring discordant genotypes likely represent vestiges of interbreeding events that occurred in the past. Based solely on diagnostic polymorphisms in COI and *Tpi* regions, MA02 and AR03 populations showed increased levels of strain hybridization, and we were able to describe the level of gene flow of these locations based on GBS data set.

GBS data revealed high levels of gene flow and low genetic differentiation between MA02 and RS population, which was composed by pure R-strain samples. Since these two populations were the most geographically distant locations sampled in Brazil, apart over 3200 km, we presumed that MA02 was composed by R-strain specimens with recent events of hybridization. Altogether, the genetic analysis based on pairwise F_ST_ distances and PCA plots confirmed that the Brazilian populations are structured by host strains, rather than by geographical ecoregions.

Long distance migration enables FAW populations to travel from Southern Texas and Florida up to Canada, a distance of nearly 2500 km, in less than three months [3, 43]. Therefore given its strong flight performance, we can hypothesize that FAW is also performing long distance migration within Brazil sufficient to keep populations homogeneous within each host strain.

Several studies comparing populations from Brazil and Argentina in the past showed strong similarities between the two countries [4, 44–46], which would be expected if the great majority of Argentina FAW are derived from seasonal migrations from Brazil. To some extent, Brazilian and Argentinian populations featured common ancestry, and the population from Buenos Aires (AR02) had the lowest genetic differentiation with Brazilian populations.

Nevertheless, F_ST_ analysis indicated significant genetic structure between countries and among provinces inside Argentina. This result is consistent with observations of mating incompatibility between populations collected in northern Argentina compared to those from the Pampas region, which indicated pre-reproductive isolating barriers between geographically separated populations [6]. GBS data for another important Noctuidae pest in the Americas, the sugarcane borer (SCB), showed a similar pattern of genetic structure between Argentina and Brazil populations, and among populations within Argentina [47, 48]. We hypothesize that Argentina likely contains one or more endemic FAW populations that exhibit significant geographical isolation and that additional studies are required to better investigate the fine scale genetic structure of FAW as well as identify locations capable of supporting permanent FAW populations in this country.

In order to explain how selection pressures might be affecting FAW populations in South America, we examined the putative annotations of genes containing outlier SNPs. Host strain outliers were mostly concentrated on the sex chromosome Z, suggesting that the selection pressures are acting upon specific regions of the FAW genome. This result corroborates with previous study where the preponderance of strain specific SNPs were Z-linked [49], and is consistent with the proposal that strain divergence is being driven primarily by Z-chromosome functions [50]. We believe that by reducing complexity of the genome, the GBS method was able to capture a fairly large number of polymorphisms in the Z-chromosome, and thereby discriminate between the R- and C-strains. It is possible that previous research based on nuclear SSR markers [12] that did not differentiate the host strains lacked sufficient coverage of the sex chromosome.

Other functional annotations revealed proteins that were likely involved in binding activities and that were present in or related to the cell membrane. Mutations in Cry receptor genes have been reported in numerous lepidopteran species to be the most common mechanism of resistance against *Bt* toxins [51], and here we found outlier SNPs in genes likely coding for Cry receptors such as GPI-anchored glycoprotein, cadherin and zinc carboxypeptidase. We also found outlier SNPs in genes possibly coding many important enzymes such as cytochrome P450 CYP314A1 [52], esterase FE4-like [53], JHAMT [54], and also proteins related to cuticle and chitin, which may be an indication of response to management with insecticides [55]. Two noteworthy outlier SNPs associated with host strains were in genes possibly encoding an odorant receptor and a UDP-glucuronosyltransferase. Odorant receptors function in insects olfaction process, which is indispensable for host selection for feeding and oviposition [54]. UDP-glucuronosyltransferase, in turn, appears to be associated with C-strain ability to detoxify DIMBOA [56], a toxic compound produced by corn plants but not rice. In conclusion, our work strongly suggest that positive selection is affecting allele frequencies at the level of populations and host strains.

From the Insect Resistance Management (IRM) perspective, resistance evolution is one of the most challenging problems in the sustainable control of FAW [57]. Therefore understanding patterns of gene flow and consequent risks for spread of field-evolved resistance alleles are crucial for effective management. Our GBS data set poses a challenging scenario in Brazil, where locations presented high levels of gene flow across all ecoregions and low genetic structure within host strains. Moreover, pairwise F_ST_ distances showed genetic structure between FAW populations of Brazil and Argentina, which has important IRM implications if resistant populations are reported in either country.

In conclusion, by combining classic molecular markers for FAW host strain identification, and genome-wide SNPs identified with GBS, we obtained more resolution of population structure than previously reported. The genetic structure and pattern of FAW in Argentina and Brazil reinforces the importance of phytosanitary barriers between countries for effective FAW management in each location. In agreement with this issue, outlier analysis suggested that positive selection is associated with field management and host strain divergences. Taking all this into consideration, current GBS data proved to be useful for population genomics research in South America and it may be applied to other geographies where the species has been introduced.

## Supporting information

S1 Table Genetic diversity estimates of fall armyworm (*Spodoptera frugiperda*) populations from 15 locations of Brazil and Argentina estimated from 3309 SNP loci. Most populations were not found at equilibrium. Brazilian populations featured lower observed heterozygosity than the expected at p < 0.05. The coefficient FIS also indicated that the Brazilian populations collected in corn fields had significant inbreeding. On the other hand, the fall armyworm populations from Argentina featured more outbreeding and private alleles.
(DOCX)

S2 Table Gene Ontology (GO) biological process description for outlier loci under positive selection. *associated with populations. **associated with host strains and populations. Loci with no mark are associated with host strains.
(DOCX)

S3 Table Outlier loci under positive selection with blast hits in NCBI with > 90% similarity. *associated with populations. **associated with host strains and populations. Loci with no mark are associated with host strains.
(DOCX)

S4 Table Geographic distance matrix showing the straight-line distances (Km) between locations. Darker orange indicates longer distances.
(DOCX)

S1 Fig Pairwise FST for fall armyworm sampling locations. (A) FST calculated with all variant loci (3309 SNPs). (B) FST calculated using neutral markers (2853 SNPs). (C) FST was calculated using candidates putatively under positive selection (456 SNPs) obtained by three methods (FLK, PCAdapt, FstHet). Darker green color represents a higher degree of differentiation.
(TIF)

S2 Fig Detection of outlier SNPs under positive selection using Pcadapt, FstHet, and FLK. The Venn diagrams shows the number of outlier SNPs associated to (A) FAW host strains and (B) populations.
(TIF)

S3 Fig Cross-validation method implemented in ADMIXTURE v1.3.0 to estimate the number of ancestral populations (k) inferred from (A) neutral markers and (B) outlier markers. (TIF)

S4 Fig Discriminant Analysis of Principal Components (DAPC) scatterplot showing the first two linear discriminants. Geographic locations are represented by different colors, and dots represent different individuals. The inset shows BIC values for different number of k clusters. Analysis performed with (A) All SNP loci, (B) 2,853 neutral SNPs, (C) 456 outliers. Plots generated using adegenet package for R software. Sampling locations were considered as priori groupings.
(TIF)

S5 Fig Polymorphisms frequencies in candidate genes under selection in Brazilian locations. Locations were represented mostly by C-strain moths, except for MA02 and RS locations where most samples were identified as R-strain.
(TIF)

## References

[1] Montezano DG, Specht A, Sosa-Gómez DR, Roque-Specht VF, Sousa-Silva JC, Paula-Moraes SV, et al. Host plants of *Spodoptera frugiperda* (Lepidoptera: Noctuidae) in the Americas. Afr Entomol. 2018; 26(2):286–300. doi: 10.4001/003.026.0286

[2] Gutiérrez-Moreno R, Mota-Sanchez D, Blanco CA, Whalon ME, Terán-Santofimio H, Rodriguez-Maciel JC, et al. Field-Evolved Resistance of the Fall Armyworm (Lepidoptera: Noctuidae) to Synthetic Insecticides in Puerto Rico and Mexico. J Econ Entomol. 2019;112: 792–802. doi: 10.1093/jee/toy372 30535077

[3] Westbrook JK, Nagoshi RN, Meagher RL, Fleischer SJ, Jairam S. Modeling seasonal migration of fall armyworm moths. Int J Biometeorol. 2016; 60(2):255–67. doi: 10.1007/s00484-015-1022-x 26045330

[4] Nagoshi RN, Vizuete JLA, Murúa MG, Garcés-Carrera S. Comparisons of fall armyworm haplotypes between the Galápagos Islands and mainland Ecuador indicate limited migration to and between islands. Sci Rep. 2021;11: 3457. doi: 10.1038/s41598-021-83111-5 33568766 PMC7875964

[5] Timilsena BP, Niassy S, Kimathi E, Abdel-Rahman EM, Seidl-Adams I, Wamalwa M, et al. Potential distribution of fall armyworm in Africa and beyond, considering climate change and irrigation patterns. Sci Rep. 2022;12: 539. doi: 10.1038/s41598-021-04369-3 35017586 PMC8752590

[6] Murúa MG, Vera MT, Abraham S, Juaréz ML, Prieto S, Head GP, et al. Fitness and mating compatibility of *Spodoptera frugiperda* (Lepidoptera: Noctuidae) populations from different host plant species and regions in Argentina. Ann Entomol Soc Am. 2008;101: 639–649. doi: 10.1603/0013-8746(2008)101[639:FAMCOS]2.0.CO;2

[7] Pashley DP. Host-associated Genetic Differentiation in Fall Armyworm (Lepidoptera: Noctuidae): A Sibling Species Complex? Ann Entomol Soc Am. 1986;79: 898–904. doi: 10.1093/aesa/79.6.898

[8] Pashley DP, Martin JA. Reproductive Incompatibility Between Host Strains of the Fall Armyworm (Lepidoptera: Noctuidae). Ann Entomol Soc Am. 1987;80: 731–733. doi: 10.1093/aesa/80.6.731

[9] Levy HC, Garcia-Maruniak A, Maruniak JE. Strain identification of *Spodoptera frugiperda* (Lepidoptera: Noctuidae) insects and cell line: PCR-RFLP of cytochrome oxidase C subunit I gene. Florida Entomol. 2003;85: 186–190. doi: 10.1653/0015-4040(2002)085[0186:SIOSFL]2.0.CO;2

[10] Nagoshi RN. The fall armyworm triose phosphate isomerase (Tpi) gene as a marker of strain identity and interstrain mating. Ann Entomol Soc Am. 2010;103: 283–292. doi: 10.1603/An09046

[11] Silva-Brandão KL, Peruchi A, Seraphim N, Murad NF, Carvalho RA, Farias JR, et al. Loci under selection and markers associated with host plant and host-related strains shape the genetic structure of Brazilian populations of *Spodoptera frugiperda* (Lepidoptera, Noctuidae). PLoS One. 2018;13: 1–28. doi: 10.1371/journal.pone.0197378 . 29787608 PMC5963752

[12] Arias O, Cordeiro EMG, Corrêa AS, Domingues FA, Guidolin AS, Omoto C. Population genetic structure and demographic history of *Spodoptera frugiperda* (Lepidoptera: Noctuidae): implications for insect resistance management programs. Pest Manag Sci. 2019;75: 2948–2957. doi: 10.1002/ps.5407 30868715

[13] Martinelli S, Barata RM, Zucchi MI, De Castro Silva-Filho M, Omoto C. Molecular variability of *Spodoptera frugiperda* (Lepidoptera: Noctuidae) populations associated to maize and cotton crops in Brazil. J Econ Entomol. 2006;99: 519–526. doi: 10.1093/jee/99.2.519 16686155

[14] Cheng J, Kao H, Dong S. Population genetic structure and gene flow of rare and endangered *Tetraena mongolica* Maxim. revealed by reduced representation sequencing. BMC Plant Biol. 2020;20: 391. doi: 10.1186/s12870-020-02594-y . 32842966 PMC7448513

[15] Doyle JJ, Doyle JL. A rapid DNA isolation procedure for small quantities of fresh leaf tissue. Phytochemical Bulletin. 1987. pp. 11–15.

[16] Thompson JD, Higgins DG, Gibson TJ. CLUSTAL W: improving the sensitivity of progressive multiple sequence alignment through sequence weighting, position-specific gap penalties and weight matrix choice. Nucleic Acids Res. 1994;22: 4673–4680. doi: 10.1093/nar/22.22.4673 . 7984417 PMC308517

[17] Kumar S, Stecher G, Tamura K. MEGA7: Molecular Evolutionary Genetics Analysis Version 7.0 for Bigger Datasets. Mol Biol Evol. 2016;33: 1870–1874. doi: 10.1093/molbev/msw054 . 27004904 PMC8210823

[18] Poland JA, Brown PJ, Sorrells ME, Jannink JL. Development of high-density genetic maps for barley and wheat using a novel two-enzyme genotyping-by-sequencing approach. PLoS One. 2012;7. doi: 10.1371/journal.pone.0032253 . 22389690 PMC3289635

[19] Andrews S. FASTQC. A quality control tool for high throughput sequence data. 2010. https://www.bioinformatics.babraham.ac.uk/projects/fastqc/

[20] Catchen JM, Amores A, Hohenlohe P, Cresko W, Postlethwait JH. Stacks: Building and Genotyping Loci De Novo From Short-Read Sequences. G3 Genes|Genomes|Genetics. 2011;1: 171 LP– 182. doi: 10.1534/g3.111.000240 . 22384329 PMC3276136

[21] Xiao H, Ye X, Xu H, Mei Y, Yang Y, Chen X, et al. The genetic adaptations of fall armyworm *Spodoptera frugiperda* facilitated its rapid global dispersal and invasion. Mol Ecol Resour. 2020;20: 1050–1068. doi: 10.1111/1755-0998.13182 32359007

[22] Li H. Aligning sequence reads, clone sequences and assembly contigs with BWA-MEM. arXiv Prepr arXiv. 2013; 0(0):3.

[23] Li H, Handsaker B, Wysoker A, Fennell T, Ruan J, Homer N, et al. The Sequence Alignment/Map format and SAMtools. Bioinformatics. 2009;25: 2078–2079. doi: 10.1093/bioinformatics/btp352 . 19505943 PMC2723002

[24] Garrison E, Marth G. Haplotype-based variant detection from short-read sequencing. arXiv Prepr arXiv12073907. 2012; 9.

[25] Danecek P, Auton A, Abecasis G, Albers CA, Banks E, DePristo MA, et al. The variant call format and VCFtools. Bioinformatics. 2011;27: 2156–2158. doi: 10.1093/bioinformatics/btr330 . 21653522 PMC3137218

[26] Goudet J. hierfstat, a package for r to compute and test hierarchical F-statistics. Mol Ecol Notes. 2005;5: 184–186. doi: 10.1111/j.1471-8286.2004.00828.x

[27] Weir B, Cockerham C. Estimating F-Statistics for the Analysis of Population-Structure. Evolution 38: 1358–1370. Evolution (N Y). 1984;38: 1358–1370. doi: 10.1111/j.1558-5646.1984.tb05657.x 28563791

[28] Dray S, Dufour A-B. The ade4 Package: Implementing the Duality Diagram for Ecologists. J Stat Softw. 2007;22: 1–20. doi: 10.18637/jss.v022.i04

[29] Jombart T, Devillard S, Balloux F. Discriminant analysis of principal components: a new method for the analysis of genetically structured populations. BMC Genet. 2010;11: 94. doi: 10.1186/1471-2156-11-94 . 20950446 PMC2973851

[30] Jombart T, Ahmed I. adegenet 1.3–1: new tools for the analysis of genome-wide SNP data. Bioinformatics. 2011;27: 3070–3071. doi: 10.1093/bioinformatics/btr521 . 21926124 PMC3198581

[31] R Core Team. A Language and Environment for Statistical Computing. R Foundation for Statistical Computing, Vienna, Austria. 2016. http://www.R-project.org/

[32] Alexander DH, Novembre J, Lange K. Fast model-based estimation of ancestry in unrelated individuals. Genome Res. 2009;19: 1655–1664. doi: 10.1101/gr.094052.109 . 19648217 PMC2752134

[33] Flanagan SP, Jones AG. Constraints on the FST-Heterozygosity Outlier Approach. J Hered. 2017;108: 561–573. doi: 10.1093/jhered/esx048 28486592

[34] Luu K, Bazin E, Blum MGB. pcadapt: an R package to perform genome scans for selection based on principal component analysis. Mol Ecol Resour. 2017;17: 67–77. doi: 10.1111/1755-0998.12592 27601374

[35] Bonhomme M, Chevalet C, Servin B, Boitard S, Abdallah J, Blott S, et al. Detecting selection in population trees: the Lewontin and Krakauer test extended. Genetics. 2010;186: 241–262. doi: 10.1534/genetics.104.117275 . 20855576 PMC2940290

[36] Götz S, García-Gómez JM, Terol J, Williams TD, Nagaraj SH, Nueda MJ, et al. High-throughput functional annotation and data mining with the Blast2GO suite. Nucleic Acids Res. 2008;36: 3420–3435. doi: 10.1093/nar/gkn176 . 18445632 PMC2425479

[37] Ye J, Zhang Y, Cui H, Liu J, Wu Y, Cheng Y, et al. WEGO 2.0: a web tool for analyzing and plotting GO annotations, 2018 update. Nucleic Acids Res. 2018;46: W71–W75. doi: 10.1093/nar/gky400 . 29788377 PMC6030983

[38] Zucchi MI, Cordeiro E, Allen C, Novello M, Viana JPG, Brown PJ, et al. Patterns of Genome-Wide Variation, Population Differentiation and SNP Discovery of the Red Banded Stink Bug (*Piezodorus guildinii*). Sci Rep. 2019;9: 14480. doi: 10.1038/s41598-019-50999-z . 31597944 PMC6785548

[39] Zucchi MI, Cordeiro EMG, Wu X, Lamana LM, Brown PJ, Manjunatha S, et al. Population genomics of the neotropical brown stink bug, *Euschistus heros*: The most important emerging insect pest to soybean in Brazil. Front Genet. 2019;10: 1035. doi: 10.3389/fgene.2019.01035 . 31749834 PMC6844245

[40] Bergamo LW, Silva-Brandão KL, Vicentini R, Fresia P, Azeredo-Espin AML. Genetic Differentiation of a New World Screwworm Fly Population from Uruguay Detected by SNPs, Mitochondrial DNA and Microsatellites in Two Consecutive Years. Insects. 2020;11: 539. doi: 10.3390/insects11080539 . 32824385 PMC7469150

[41] Anderson CJ, Tay WT, McGaughran A, Gordon K, Walsh TK. Population structure and gene flow in the global pest, *Helicoverpa armigera*. Mol Ecol. 2016;25: 5296–5311. doi: 10.1111/mec.13841 . 27661785

[42] Cordeiro EMG, Pantoja-Gomez LM, de Paiva JB, Nascimento ARB, Omoto C, Michel AP, et al. Hybridization and introgression between *Helicoverpa armigera* and *H*. *zea*: an adaptational bridge. BMC Evol Biol. 2020;20: 61. doi: 10.1186/s12862-020-01621-8 . 32450817 PMC7249340

[43] Nagoshi RN, Goergen G, Koffi D, Agboka K, Adjevi AKM, Du Plessis H, et al. Genetic studies of fall armyworm indicate a new introduction into Africa and identify limits to its migratory behavior. Sci Rep. 2022;12: 1941. Epub 20220204. doi: 10.1038/s41598-022-05781-z . 35121788 PMC8816908

[44] Clark PL, Molina-Ochoa J, Martinelli S, Skoda SR, Isenhour DJ, Lee DJ, et al. Population variation of the fall armyworm, *Spodoptera frugiperda*, in the Western Hemisphere. J Insect Sci. 2007;7: 1–10. doi: 10.1673/031.007.0501 . 20334595 PMC2999398

[45] Nagoshi RN, Rosas-García NM, Meagher RL, Fleischer SJ, Westbrook J, Sappington TW, et al. Haplotype profile comparisons between *Spodoptera frugiperda* (Lepidoptera: Noctuidae) populations from mexico with those from Puerto Rico, South America, and the United States and their implications to migratory behavior. J Econ Entomol. 2015;108: 135–144. doi: 10.1093/jee/tou044 26470113

[46] Schlum KA, Lamour K, de Bortoli CP, Banerjee R, Meagher R, Pereira E, et al. Whole genome comparisons reveal panmixia among fall armyworm (*Spodoptera frugiperda*) from diverse locations. BMC Genomics. 2021;22: 179. Epub 20210312. doi: 10.1186/s12864-021-07492-7 . 33711916 PMC7953542

[47] Fogliata S V, Perera MF, Alves-Pereira A, Zucchi MI, Murúa MG. Unraveling the population structure of the sugarcane borer, *Diatraea saccharalis*, in Argentina. Entomol Exp Appl. 2022;170: 530–545. doi: 10.1111/eea.13174

[48] Francischini FJB, Cordeiro EMG, de Campos JB, Alves-Pereira A, Viana JPG, Wu X, et al. *Diatraea saccharalis* history of colonization in the Americas. The case for human-mediated dispersal. PLoS One. 2019;14: e0220031. doi: 10.1371/journal.pone.0220031 31339922 PMC6656350

[49] Tessnow AE, Raszick TJ, Porter P, Sword GA. Patterns of genomic and allochronic strain divergence in the fall armyworm, *Spodoptera frugiperda* (J.E. Smith). Ecol Evol. 2022;12: e8706. Epub 20220321. doi: 10.1002/ece3.8706 . 35356552 PMC8938225

[50] Prowell DP. Sex linkage and speciation in Lepidoptera. In: Howard D, Berlocher S, editors. Endless forms: Species and speciation. New York: Oxford Press; 1998. p. 309–19.

[51] Peterson B, Bezuidenhout CC, Van den Berg J. An Overview of Mechanisms of Cry Toxin Resistance in Lepidopteran Insects. J Econ Entomol. 2017;110: 362–377. doi: 10.1093/jee/tow310 28334065

[52] Shu B, Yu H, Li Y, Zhong H, Li X, Cao L, et al. Identification of azadirachtin responsive genes in *Spodoptera frugiperda* larvae based on RNA-seq. Pestic Biochem Physiol. 2021;172: 104745. doi: 10.1016/j.pestbp.2020.104745 33518039

[53] Boaventura D, Buer B, Hamaekers N, Maiwald F, Nauen R. Toxicological and molecular profiling of insecticide resistance in a Brazilian strain of fall armyworm resistant to Bt Cry1 proteins. Pest Manag Sci. 2020/09/10. 2021;77: 3713–3726. doi: 10.1002/ps.6061 32841530 PMC8359450

[54] Guo P, Zhang Y, Zhang L, Xu H, Zhang H, Wang Z, et al. Structural basis for juvenile hormone biosynthesis by the juvenile hormone acid methyltransferase. J Biol Chem. 2021;297: 101234. doi: 10.1016/j.jbc.2021.101234 34562453 PMC8526772

[55] Zhu J-Y, Li L, Xiao K-R, He S-Q, Gui F-R. Genomic and Transcriptomic Analysis Reveals Cuticular Protein Genes Responding to Different Insecticides in Fall Armyworm *Spodoptera frugiperda*. Insects. 2021;12. doi: 10.3390/insects12110997 34821798 PMC8622913

[56] Silva-Brandão KL, Murad NF, Peruchi A, Martins CHZ, Omoto C, Figueira A, et al. Transcriptome differential co-expression reveals distinct molecular response of fall-armyworm strains to DIMBOA. Pest Manag Sci. 2021;77: 518–526. doi: 10.1002/ps.6051 32815313

[57] Zhang L, Liu B, Zheng W, Liu C, Zhang D, Zhao S, et al. Genetic structure and insecticide resistance characteristics of fall armyworm populations invading China. Mol Ecol Resour. 2020;20: 1682–1696. doi: 10.1111/1755-0998.13219 32619331 PMC7689805

