# Supplementary material for: Population genomics of fall armyworm by genotyping-by-sequencing: Implications for pest management
Source: PLoS One. 2023 Apr 18;18(4):e0284587. doi: 10.1371/journal.pone.0284587 (PMC10112782; doi:10.1371/journal.pone.0284587)
Supplement: S1 Table — Most populations were not found at equilibrium. Brazilian populations featured lower observed heterozygosity than the expected at p < 0.05. The coefficient FIS also indicated that the Brazilian populations collected in corn fields had significant inbreeding. On the other hand, the fall armyworm populations from Argentina featured more outbreeding and private alleles. (DOCX) [file pone.0284587.s001.docx]

**S1 Table. Genetic diversity estimates of fall armyworm (Spodoptera frugiperda) populations from 15 locations of Brazil and Argentina estimated from 3309 SNP loci.** **Most populations were not found at equilibrium. Brazilian populations featured lower observed heterozygosity than the expected at p < 0.05. The coefficient F_IS_ also indicated that the Brazilian populations collected in corn fields had significant inbreeding. On the other hand, the fall armyworm populations from Argentina featured more outbreeding and private alleles.**

| Population | *Ap* | %P | *H_O_* | *H_E_* | *π* | *F_IS_* |
| --- | --- | --- | --- | --- | --- | --- |
| BA02 | 0 | 52.3 | 0.0609* | 0.0650 | 0.0669 ± 0.0017 | 0.03 |
| BA03 | 0 | 54.1 | 0.0616* | 0.0660 | 0.0679 ± 0.0017 | 0.03* |
| DF | 0 | 55.6 | 0.0620* | 0.0674 | 0.0692 ± 0.0017 | 0.04* |
| GO | 0 | 61.5 | 0.0600* | 0.0668 | 0.0683 ± 0.0016 | 0.05* |
| MT01 | 1 | 60.2 | 0.0594* | 0.0677 | 0.0692 ± 0.0017 | 0.06* |
| MT02 | 0 | 56.6 | 0.0593* | 0.0657 | 0.0674 ± 0.0017 | 0.05* |
| SP | 1 | 59.4 | 0.0586* | 0.0666 | 0.0681 ± 0.0017 | 0.07* |
| PR | 0 | 35.3 | 0.0604 | 0.0636 | 0.0676 ± 0.0020 | 0.03* |
| MA01 | 0 | 38.3 | 0.0593* | 0.0662 | 0.0699 ± 0.0020 | 0.04* |
| MA02 | 9 | 39.6 | 0.0616* | 0.0715 | 0.0748 ± 0.0021 | 0.04* |
| RS | 0 | 13.0 | 0.0524* | 0.0451 | 0.0571 ± 0.0028 | 0.01 |
| SC | 0 | 45.5 | 0.0602* | 0.0640 | 0.0665 ± 0.0018 | 0.03 |
| AR01 | 46 | 22.0 | 0.0887* | 0.0688 | 0.0723 ± 0.0027 | -0.03 |
| AR02 | 1 | 9.3 | 0.0498* | 0.0375 | 0.0451 ± 0.0025 | -0.01 |
| AR03 | 49 | 20.9 | 0.0611 | 0.0618 | 0.0641 ± 0.0025 | 0.02 |

*Significant values (p < 0.05).

Ap: number of private alleles; %P: percentage of polymorphic loci; H_O_: observed heterozygosity; H_E_: expected heterozygosity; *π*: nucleotide diversity ± standard error; F_IS_: inbreeding coefficient.
