# Supplementary material for: Population genomics of fall armyworm by genotyping-by-sequencing: Implications for pest management
Source: PLoS One. 2023 Apr 18;18(4):e0284587. doi: 10.1371/journal.pone.0284587 (PMC10112782; doi:10.1371/journal.pone.0284587)
Supplement: S2 Table — *associated with populations. **associated with host strains and populations. Loci with no mark are associated with host strains. (DOCX) [file pone.0284587.s002.docx]

**S2 Table. Gene Ontology (GO) biological process description for outlier loci under positive selection. *associated with populations. **associated with host strains and populations. Loci with no mark are associated with host strains.**

| **Loci** | **Similar to** | **e-Value** | **Mean similarity (%)** | **GO description** |
| --- | --- | --- | --- | --- |
| SNP_18** | zinc-type alcohol dehydrogenase-like protein C1773.06c | 0 | 98.53 | Oxidoreductases |
| SNP_29 | solute carrier family 35 member C2 | 0 | 95.94 | integral component of membrane |
| SNP_41** | dynein heavy chain 5, axonemal isoform X2 | 0 | 98.06 | microtubule-based movement |
| SNP_67 | UNC93-like protein | 0 | 99.49 | regulation of potassium ion transport |
| SNP_96 | insulin-like growth factor 2 mRNA-binding protein 1 isoform X2 | 0 | 99.48 | mRNA transport |
| SNP_110 | carboxyl-terminal PDZ ligand of neuronal nitric oxide synthase protein-like isoform X3 | 0 | 97.17 | localization |
| SNP_127** | epithelial discoidin domain-containing receptor 1-like isoform X1 | 0 | 98.91 | peptidyl-tyrosine phosphorylation |
| SNP_137** | lysophospholipid acyltransferase 5-like | 0 | 98.76 | lipid modification |
| SNP_150 | alpha-mannosidase 2-like | 0 | 99.22 | mannose metabolic process |
| SNP_160 | hepatic leukemia factor-like isoform X10 | 2.4E-156 | 95.46 | regulation of transcription by RNA polymerase II |
| SNP_162 | ATP synthase subunit gamma, mitochondrial-like | 0 | 93.41 | ATP synthesis coupled proton transport |
| SNP_184** | protein bric-a-brac 1-like isoform X1 | 0 | 97.29 | eye-antennal disc morphogenesis |
| SNP_230 | ras-related protein Rab6 isoform X1 | 1.9E-156 | 100 | GTPase activity |
| SNP_240* | protein eiger | 0 | 93.07 | immune response |
| SNP_262 | electron transfer flavoprotein subunit alpha, mitochondrial | 0 | 99.7 | electron transport chain |
| SNP_264 | alkaline ceramidase | 0 | 98.74 | ceramide metabolic process |
| SNP_333* | cullin-associated NEDD8-dissociated protein 1 | 0 | 98.41 | SCF complex assembly |
| SNP_365* | RNA-binding protein 24-B-like isoform X1 | 5.08E-93 | 94.7 | RNA binding |
| SNP_421 | odorant receptor 46a-like | 0 | 98.95 | detection of chemical stimulus involved in sensory perception of smell |
| SNP_574 | protein obstructor-E isoform X2 | 6.6E-167 | 99.32 | chitin binding |
| SNP_582 | ras-related protein Rab-9B | 2.5E-173 | 99.21 | GTPase activity |
| SNP_599 | replication factor C subunit 1-like | 0 | 93.49 | DNA replication |
| SNP_648* | GTPase-activating protein | 0 | 96.36 | regulation of GTPase activity |
| SNP_677 | filamin-C isoform X1 | 0 | 97.79 | actin cytoskeleton organization |
| SNP_682 | aquaporin AQPAe.a-like | 3.5E-158 | 97.76 | transmembrane transport |
| SNP_883 | methylglutaconyl-CoA hydratase, mitochondrial-like | 0 | 98.59 | catalytic activity |
| SNP_925 | ER lumen protein-retaining receptor | 9.8E-132 | 99.43 | protein retention in ER lumen |
| SNP_1055* | ABC transporter C family member 13 | 0 | 96.52 | transmembrane transport |
| SNP_1060** | exportin-1 | 0 | 99.02 | protein export from nucleus |
| SNP_1104* | kinesin-like protein CG14535 isoform X1 | 0 | 97.94 | microtubule-based movement |
| SNP_1137* | transcription factor sem-2-like isoform X1 | 5.8E-159 | 99.5 | DNA binding |
| SNP_1208 | guanine nucleotide exchange factor subunit Rich-like isoform X4 | 0 | 98.85 | intracellular protein transport |
| SNP_1209* | cuticle protein 19-like | 1.75E-97 | 90.68 | structural constituent of cuticle |
| SNP_1215 | uncharacterized protein LOC118265954 | 0 | 92.4 | protein transport |
| SNP_1249 | disintegrin and metalloproteinase domain-containing protein 11 | 0 | 97.07 | proteolysis |
| SNP_1263* | RNA-binding protein Musashi homolog Rbp6 isoform X2 | 0 | 96.32 | RNA binding |
| SNP_1291* | CAD protein isoform X2 | 0 | 95.89 | carbohydrate metabolic process |
| SNP_1391 | peroxisomal acyl-coenzyme A oxidase 3 | 0 | 96.11 | fatty acid beta-oxidation |
| SNP_1428 | semaphorin-2A isoform X2 | 0 | 99.34 | cell differentiation |
| SNP_1434* | chymotrypsin-like serine protease precursor | 0 | 94.27 | proteolysis |
| SNP_1513* | uncharacterized protein LOC118267576 | 1.6E-113 | 96.59 | integral component of membrane |
| SNP_1559* | esterase FE4-like | 0 | 98.12 | isoprenoid biosynthetic process |
| SNP_1568 | decaprenyl-diphosphate synthase subunit 1 | 0 | 97.89 | isoprenoid biosynthetic process |
| SNP_1595* | uncharacterized protein LOC110373722 isoform X1 | 6.49E-97 | 98.89 | integral component of membrane |
| SNP_1654 | lissencephaly-1 homolog | 0 | 99.61 | microtubule sliding; cell division |
| SNP_1699* | unconventional myosin ID | 0 | 94.73 | cytoskeletal motor activity |
| SNP_1714 | uncharacterized protein LOC118269482 | 0 | 94.18 | integral component of membrane |
| SNP_1749 | receptor-type guanylate cyclase gcy-1 | 0 | 97.7 | cGMP biosynthetic process |
| SNP_1780 | mothers against decapentaplegic homolog 6 | 0 | 98.37 | regulation of developmental process |
| SNP_1823* | homeotic protein Sex combs reduced-like | 0 | 97.52 | regulation of transcription by RNA polymerase II |
| SNP_1831 | homeotic protein antennapedia | 7.2E-158 | 99.54 | regulation of transcription by RNA polymerase II |
| SNP_1853 | cuticular protein lCPAP1-B | 9.2E-116 | 95.75 | chitin binding |
| SNP_1949 | octopamine receptor beta-3R-like | 0 | 99.71 | G protein-coupled receptor signaling pathway |
| SNP_1957* | complexin isoform X1 | 3.66E-28 | 100 | neurotransmitter transport |
| SNP_1977* | cyclin-dependent kinase inhibitor 1-like isoform X2 | 1.3E-140 | 94.97 | negative regulation of cyclin-dependent protein serine/threonine kinase activity |
| SNP_1997 | dynein heavy chain 1, axonemal-like | 0 | 95.8 | microtubule-based movement |
| SNP_2124 | uncharacterized protein LOC118271900 | 0 | 95.99 | signal transduction |
| SNP_2183* | uncharacterized protein LOC118271592 | 0 | 97.25 | positive regulation of protein tyrosine kinase activity |
| SNP_2213* | transmembrane protein 184B isoform X1 | 0 | 97.45 | integral component of membrane |
| SNP_2301* | probable kinetochore protein nuf2 isoform X2 | 0 | 99.11 | regulation of microtubule polymerization or depolymerization |
| SNP_2355** | protein kinase DC2 | 0 | 99.94 | protein phosphorylation |
| SNP_2359* | bcl-2-related ovarian killer protein homolog A-like | 3.3E-134 | 99.73 | regulation of apoptotic process |
| SNP_2374 | phosphatidylserine synthase 1-like | 0 | 98.59 | phosphatidylserine biosynthetic process |
| SNP_2396 | dopa decarboxylase | 0 | 99.71 | cellular amino acid metabolic process |
| SNP_2399 | E3 ubiquitin-protein ligase MIB1-like | 0 | 99.07 | Notch signaling pathway; protein ubiquitination |
| SNP_2407* | platelet-activating factor acetylhydrolase IB subunit beta homolog | 2.5E-169 | 99.38 | oogenesis |
| SNP_2526* | tumor necrosis factor alpha-induced protein 8-like protein isoform X1 | 1.4E-137 | 99.53 | regulation of apoptotic process |
| SNP_2532* | phorbol ester/diacylglycerol-binding protein unc-13-like | 9.88E-86 | 98.8 | chemical synaptic transmission |
| SNP_2552 | A disintegrin and metalloproteinase with thrombospondin motifs 7 | 0 | 95.29 | integrin-mediated signaling pathway |
| SNP_2659* | homeobox protein cut isoform X7 | 0 | 97.62 | cell development |
| SNP_2700 | CLIP domain-containing serine protease 2-like isoform X1 | 0 | 95.13 | proteolysis |
| SNP_2719 | N-alpha-acetyltransferase 40 | 0 | 94.34 | histone acetylation |
| SNP_2720 | DNA polymerase alpha subunit B-like | 0 | 98.08 | DNA replication |
| SNP_2763 | neurexin-1-like isoform X2 | 0 | 99.02 | anatomical structure morphogenesis |
| SNP_2792* | uncharacterized protein LOC118276239 | 0 | 98.63 | proteolysis |
| SNP_2799* | uncharacterized protein LOC118276304 | 0 | 94.68 | chitin binding |
| SNP_2804* | spondin-2 isoform X1 | 0 | 97.27 | cell adhesion |
| SNP_2809 | phospholipase D3-like isoform X1 | 0 | 98.1 | oogenesis |
| SNP_2863** | endoribonuclease Dicer-like | 0 | 94.14 | production of miRNAs involved in gene silencing by miRNA |
| SNP_2876 | RNA/RNP complex-1-interacting phosphatase-like | 9E-161 | 92.91 | protein dephosphorylation |
| SNP_2948 | irregular chiasm C-roughest protein-like isoform X2 | 0 | 96.03 | compound eye development |
| SNP_2960 | non-canonical poly(A) RNA polymerase protein Trf4-1-like | 0 | 98.54 | RNA polyadenylation |
| SNP_3034 | fibroblast growth factor 22 isoform X1 | 2.2E-142 | 99.25 | signal transduction |
| SNP_3069* | mothers against decapentaplegic homolog 4 isoform X3 | 0 | 94.75 | regulation of developmental process |
| SNP_3074 | dopamine receptor 2-like | 0 | 98.23 | G protein-coupled receptor signaling pathway |
| SNP_3077 | cytochrome P450 CYP314A1 | 0 | 99.54 | ecdysteroid biosynthesis |
| SNP_3101 | neuronal PAS domain-containing protein 4B-like | 0 | 98.96 | positive regulation of transcription by RNA polymerase II |
| SNP_3112* | neurogenic locus protein delta | 0 | 95.44 | Notch signaling pathway |
| SNP_3139* | cuticular protein SlCPAP1-J | 0 | 98.19 | chitin binding |
| SNP_3180* | presequence protease, mitochondrial | 0 | 96.71 | proteolysis |
| SNP_3192* | uncharacterized protein LOC118279893 | 0 | 94.4 | integral component of membrane |
| SNP_3208 | putative fatty acyl-CoA reductase CG5065 | 0 | 97.38 | lipid metabolic process |
| SNP_3227 | anoctamin-4-like isoform X1 | 0 | 98.48 | protein dimerization |
| SNP_3271* | proline-, glutamic acid- and leucine-rich protein 1-like | 0 | 99.18 | regulation of transcription, DNA-templated |
