# Supplementary material for: Population genomics of fall armyworm by genotyping-by-sequencing: Implications for pest management
Source: PLoS One. 2023 Apr 18;18(4):e0284587. doi: 10.1371/journal.pone.0284587 (PMC10112782; doi:10.1371/journal.pone.0284587)
Supplement: S3 Table — *associated with populations. **associated with host strains and populations. Loci with no mark are associated with host strains. (DOCX) [file pone.0284587.s003.docx]

**S3 Table. Outlier loci under positive selection with blast hits in NCBI with > 90% similarity. *associated with populations. **associated with host strains and populations. Loci with no mark are associated with host strains.**

| **Loci** | **Similar to** | **e-Value** | **Mean similarity (%)** |
| --- | --- | --- | --- |
| SNP_44 | uncharacterized protein LOC118266193 | 0 | 97.89 |
| SNP_46** | transient receptor potential cation channel subfamily A member 1-like isoform X8 | 0 | 99.28 |
| SNP_49 | protein apterous-like isoform X1 | 0 | 90.7 |
| SNP_51 | E3 ubiquitin-protein ligase RBBP6 isoform X2 | 0 | 96.12 |
| SNP_53** | epidermal growth factor receptor isoform X2 | 0 | 98.83 |
| SNP_58 | activin receptor type-1C-like isoform X3 | 0 | 95.24 |
| SNP_64** | uncharacterized protein LOC118264930 | 0 | 95.95 |
| SNP_66 | uncharacterized protein LOC118261817 isoform X1 | 0 | 100 |
| SNP_70 | cyclic nucleotide-gated cation channel subunit A-like isoform X1 | 0 | 98.49 |
| SNP_77** | uncharacterized protein LOC118268191 isoform X1 | 0 | 98.67 |
| SNP_78 | nuclear receptor subfamily 2 group E member 1-like | 0 | 99.37 |
| SNP_81* | SCY1-like protein 2 isoform X1 | 0 | 98.41 |
| SNP_95** | eukaryotic translation initiation factor 4 gamma 3-like isoform X5 | 0 | 99.61 |
| SNP_102** | uncharacterized protein LOC118265176 | 1.9E-126 | 97.22 |
| SNP_103 | proton-coupled amino acid transporter-like protein pathetic | 0 | 99.49 |
| SNP_104 | nuclear hormone receptor FTZ-F1 | 0 | 97.86 |
| SNP_113 | protein scabrous | 0 | 94.88 |
| SNP_133* | BPTI/Kunitz domain-containing protein-like isoform X2 | 6.3E-109 | 97.22 |
| SNP_134** | T-cell acute lymphocytic leukemia protein 1 homolog isoform X6 | 2.8E-157 | 99.34 |
| SNP_135** | circadian locomoter output cycles protein kaput-like isoform X1 | 0 | 98.77 |
| SNP_147** | uncharacterized protein LOC118263576 | 0 | 93.83 |
| SNP_148 | nostrin-like isoform X2 | 0 | 99.31 |
| SNP_155** | proton-coupled amino acid transporter-like protein CG1139 isoform X1 | 0 | 99.15 |
| SNP_156 | GRB10-interacting GYF protein 2-like isoform X1 | 0 | 98.16 |
| SNP_158 | solute carrier family 35 member F6 | 0 | 95.63 |
| SNP_164 | protein furry-like isoform X1 | 0 | 99.89 |
| SNP_167 | ecdysone-induced protein 78C-like isoform X2 | 0 | 99.95 |
| SNP_171 | E3 ubiquitin-protein ligase UHRF1-like | 0 | 96.69 |
| SNP_176** | potassium voltage-gated channel protein Shaker-like isoform X1 | 0 | 98.55 |
| SNP_179* | calcium/calmodulin-dependent 3',5'-cyclic nucleotide phosphodiesterase 1-like isoform X2 | 0 | 99.02 |
| SNP_189 | chromatin complexes subunit BAP18-like isoform X4 | 3.34E-89 | 98.89 |
| SNP_190 | chondroitin sulfate proteoglycan 4-like isoform X1 | 0 | 99.51 |
| SNP_198 | coiled-coil domain-containing protein CG32809-like isoform X8 | 0 | 99.97 |
| SNP_199 | E3 ubiquitin-protein ligase MYCBP2-like isoform X13 | 0 | 99.42 |
| SNP_203** | kalirin-like isoform X1 | 0 | 98.75 |
| SNP_217** | zinc transporter 2-like isoform X2 | 0 | 99.31 |
| SNP_243* | uncharacterized protein LOC118271946 isoform X3 | 0 | 99.84 |
| SNP_249 | uncharacterized protein LOC118271979 | 0 | 97.02 |
| SNP_256 | voltage-dependent calcium channel subunit alpha-2/delta-3-like isoform X1 | 0 | 99.78 |
| SNP_274 | insulin-like receptor | 0 | 98.63 |
| SNP_304 | C-Maf-inducing protein-like | 0 | 96.52 |
| SNP_315 | protein grainyhead isoform X1 | 0 | 99.19 |
| SNP_323* | uncharacterized protein LOC118276435 | 2.3E-106 | 98.29 |
| SNP_374 | guanine nucleotide exchange factor DBS-like isoform X1 | 0 | 98.35 |
| SNP_379 | myocyte-specific enhancer factor 2 isoform X1 | 0 | 98.29 |
| SNP_396* | protein bunched, class 2/F/G isoform-like isoform X1 | 0 | 97.61 |
| SNP_423** | cytochrome P450 4d2-like isoform X1 | 0 | 98.67 |
| SNP_437* | polypyrimidine tract-binding protein 1 isoform X2 | 0 | 99.39 |
| SNP_444* | cAMP-specific 3',5'-cyclic phosphodiesterase, isoform F isoform X1 | 0 | 97.94 |
| SNP_462 | coiled-coil domain-containing protein 6-like isoform X3 | 0 | 95.55 |
| SNP_465* | hypothetical protein SFRURICE_020353 | 0 | 99.77 |
| SNP_466* | protein still life, isoform SIF type 1-like isoform X1 | 0 | 99.91 |
| SNP_477 | constitutive coactivator of PPAR-gamma-like protein 1 isoform X1 | 0 | 99.64 |
| SNP_487* | uncharacterized protein LOC118281882 isoform X2 | 0 | 97.07 |
| SNP_492** | dihydrolipoyl dehydrogenase | 0 | 97.98 |
| SNP_511* | DNA polymerase epsilon subunit 4 | 8.5E-108 | 96.19 |
| SNP_523* | akirin-like | 3.2E-106 | 94.39 |
| SNP_557* | WD repeat-containing protein 5 | 0 | 99.75 |
| SNP_563* | ecdysone-induced protein 74EF isoform X1 | 0 | 96.93 |
| SNP_617 | uncharacterized protein LOC118262780 isoform X4 | 0 | 93.72 |
| SNP_619 | probable G-protein coupled receptor No18 isoform X1 | 0 | 96.64 |
| SNP_634* | protein takeout-like | 4.8E-173 | 98.75 |
| SNP_684 | zinc finger protein DZIP1L-like | 0 | 94.49 |
| SNP_693* | asparagine-rich zinc finger protein AZF1 | 0 | 99.54 |
| SNP_719 | gamma-aminobutyric acid type B receptor subunit 1-like isoform X1 | 0 | 99.06 |
| SNP_758* | LOW QUALITY PROTEIN: synembryn-A-like | 0 | 92.15 |
| SNP_773* | proton channel OtopLc-like | 0 | 97.3 |
| SNP_798* | semaphorin-2A-like isoform X2 | 0 | 99.06 |
| SNP_816** | teneurin-a isoform X2 | 0 | 99.57 |
| SNP_892 | thrombospondin type-1 domain-containing protein 7A-like | 0 | 98.01 |
| SNP_931 | class A basic helix-loop-helix protein 15-like | 3.39E-97 | 98.95 |
| SNP_934 | protein yellow-like isoform X2 | 0 | 99.24 |
| SNP_938 | sodium-dependent nutrient amino acid transporter 1-like | 0 | 97.39 |
| SNP_941** | uncharacterized protein LOC118264104 isoform X1 | 0 | 99.31 |
| SNP_960 | heparan sulfate glucosamine 3-O-sulfotransferase 3A1 | 0 | 98.61 |
| SNP_983 | serine/threonine-protein kinase ATR-like | 0 | 98.27 |
| SNP_997 | protein winged eye-like isoform X2 | 0 | 99.24 |
| SNP_1001** | uncharacterized protein LOC118264324 | 0 | 95.15 |
| SNP_1010* | cadherin-86C isoform X1 | 0 | 98.39 |
| SNP_1021 | protein expanded-like | 0 | 99.62 |
| SNP_1024** | juvenile hormone acid O-methyltransferase-like isoform X2 | 0 | 100 |
| SNP_1034 | dachshund homolog 2 isoform X9 | 0 | 99.89 |
| SNP_1041 | uncharacterized protein LOC118265239 isoform X1 | 0 | 98.85 |
| SNP_1085** | uncharacterized protein LOC118265544 | 0 | 98.82 |
| SNP_1086 | uncharacterized protein LOC118264958 | 0 | 98.18 |
| SNP_1088 | uncharacterized protein LOC118265490 | 0 | 98.39 |
| SNP_1092 | gremlin-2-like isoform X2 | 7.8E-150 | 100 |
| SNP_1114 | CUGBP Elav-like family member 3-B isoform X1 | 0 | 98.51 |
| SNP_1127 | WD repeat-containing protein on Y chromosome-like | 0 | 96.23 |
| SNP_1149 | hemicentin-2-like isoform X1 | 0 | 99.8 |
| SNP_1195 | dystroglycan-like isoform X1 | 0 | 100 |
| SNP_1214 | sporozoite surface protein 2-like isoform X26 | 3.22E-84 | 93.27 |
| SNP_1217 | hypothetical protein SFRURICE_000691 | 0 | 95.22 |
| SNP_1219* | cerebellar degeneration-related protein 2-like isoform X3 | 0 | 99.34 |
| SNP_1233 | uncharacterized protein LOC118267214 isoform X3 | 0 | 100 |
| SNP_1239 | thioredoxin reductase 1, mitochondrial-like isoform X1 | 0 | 99.96 |
| SNP_1242* | 2-oxoglutarate dehydrogenase, mitochondrial-like isoform X2 | 0 | 98.25 |
| SNP_1283 | speract receptor-like isoform X2 | 0 | 98.54 |
| SNP_1313* | ecdysone-inducible protein E75 isoform X1 | 0 | 99.66 |
| SNP_1321 | tetratricopeptide repeat protein 37 | 0 | 98.38 |
| SNP_1325* | Ca(2+)/calmodulin-responsive adenylate cyclase-like isoform X2 | 0 | 95.75 |
| SNP_1345 | peroxisomal targeting signal 1 receptor-like | 0 | 98.26 |
| SNP_1351 | uncharacterized protein LOC118266951 | 0 | 99.27 |
| SNP_1352 | zinc carboxypeptidase-like | 0 | 96.4 |
| SNP_1358 | uncharacterized protein LOC118266809 | 0 | 98.41 |
| SNP_1361** | E3 ubiquitin-protein ligase HECW2-like isoform X2 | 0 | 98.65 |
| SNP_1371 | protein outspread-like isoform X1 | 0 | 98.95 |
| SNP_1379* | somatostatin receptor type 2-like | 0 | 99.66 |
| SNP_1474* | F-actin-monooxygenase MICAL3-like isoform X2 | 0 | 96.3 |
| SNP_1477 | proteoglycan 4-like isoform X1 | 0 | 99.34 |
| SNP_1547 | tachykinin-like peptides receptor 86C isoform X2 | 0 | 99.56 |
| SNP_1577** | uncharacterized protein LOC118268079 | 0 | 98.22 |
| SNP_1583* | uncharacterized protein LOC110374604 | 0 | 98.11 |
| SNP_1618 | titin-like | 0 | 98.47 |
| SNP_1750* | calcitonin gene-related peptide type 1 receptor-like isoform X1 | 0 | 97.74 |
| SNP_1764 | protein halfway-like isoform X2 | 0 | 98.66 |
| SNP_1767 | SPRY domain-containing SOCS box protein 3-like isoform X1 | 0 | 99.75 |
| SNP_1771 | mucin-17 isoform X1 | 0 | 97.34 |
| SNP_1772 | uncharacterized protein LOC118269350 | 1.63E-77 | 96.42 |
| SNP_1878* | protein held out wings-like isoform X3 | 0 | 98.77 |
| SNP_1967* | myotubularin-related protein 5 isoform X2 | 0 | 91.22 |
| SNP_1993* | WD repeat domain phosphoinositide-interacting protein 4-like | 0 | 95.86 |
| SNP_1999* | uncharacterized protein LOC118271498 | 0 | 97.4 |
| SNP_2011 | neurobeachin-like isoform X9 | 0 | 99.97 |
| SNP_2056 | brain tumor protein-like isoform X2 | 0 | 99.93 |
| SNP_2082 | espin-like isoform X1 | 0 | 98.6 |
| SNP_2094* | beta-1,4-N-acetylgalactosaminyltransferase bre-4-like | 0 | 97.9 |
| SNP_2099 | uncharacterized protein LOC118272254 | 0 | 99.75 |
| SNP_2127 | uncharacterized protein LOC118272157 | 0 | 91.82 |
| SNP_2132 | protein split ends-like | 0 | 97.36 |
| SNP_2146 | carboxypeptidase B-like | 3.3E-172 | 93.25 |
| SNP_2196 | low-density lipoprotein receptor-related protein 2-like isoform X1 | 0 | 99.64 |
| SNP_2229 | probable helicase senataxin | 0 | 95.05 |
| SNP_2243 | ADP-ribosylation factor-like protein 4C | 9.3E-143 | 100 |
| SNP_2272 | tyrosine-protein phosphatase Lar-like isoform X1 | 0 | 99.8 |
| SNP_2284 | facilitated trehalose transporter Tret1-like isoform X4 | 0 | 99.25 |
| SNP_2325 | sodium- and chloride-dependent glycine transporter 1-like | 0 | 98.64 |
| SNP_2326* | uncharacterized protein LOC118273335 | 1.4E-179 | 93.81 |
| SNP_2328 | protein eyes shut | 0 | 95.72 |
| SNP_2336 | tyrosine-protein kinase transmembrane receptor Ror-like isoform X2 | 0 | 97.51 |
| SNP_2345 | muscleblind-like protein 1 isoform X3 | 0 | 95.03 |
| SNP_2387 | homeotic protein spalt-major-like isoform X2 | 0 | 99.62 |
| SNP_2424* | E3 ubiquitin-protein ligase lubel-like isoform X1 | 0 | 97.53 |
| SNP_2432 | piezo-type mechanosensitive ion channel component-like isoform X7 | 0 | 98.43 |
| SNP_2449 | leucine-rich repeats and immunoglobulin-like domains protein 2 isoform X2 | 0 | 99.24 |
| SNP_2507* | sentrin-specific protease 2-like | 0 | 90.19 |
| SNP_2585* | uncharacterized protein LOC118273925 isoform X2 | 1.3E-155 | 99.27 |
| SNP_2586* | flocculation protein FLO11 | 0 | 98.6 |
| SNP_2620* | flotillin-2 isoform X2 | 0 | 99.76 |
| SNP_2622 | bromodomain-containing protein 1-like isoform X2 | 0 | 94.99 |
| SNP_2624* | LIM/homeobox protein Lhx1-like isoform X2 | 0 | 99.29 |
| SNP_2631* | mevalonate kinase | 0 | 99.47 |
| SNP_2668* | uncharacterized protein CG3556-like isoform X1 | 0 | 98.72 |
| SNP_2671 | max-binding protein MNT-like isoform X1 | 0 | 95.04 |
| SNP_2692 | RNA-binding protein Nova-2 isoform X1 | 0 | 96.56 |
| SNP_2705 | uncharacterized protein LOC111350172 | 1.34E-97 | 99.29 |
| SNP_2718 | DNA replication licensing factor Mcm5-like | 0 | 99.86 |
| SNP_2719 | UDP-glucuronosyltransferase 1-2-like | 0 | 99.38 |
| SNP_2744 | zinc finger protein 541-like isoform X1 | 0 | 98.63 |
| SNP_2774* | phosphofurin acidic cluster sorting protein 1-like isoform X1 | 0 | 98.44 |
| SNP_2798 | polycystic kidney disease protein 1-like 3 | 0 | 99.63 |
| SNP_2806 | delta-1-pyrroline-5-carboxylate synthase-like isoform X2 | 0 | 98.84 |
| SNP_2861 | protein couch potato-like | 6.6E-170 | 91.72 |
| SNP_2884 | uncharacterized protein LOC111357656 | 5.9E-66 | 98.75 |
| SNP_2921* | max dimerization protein 1-like isoform X2 | 1.6E-123 | 98.96 |
| SNP_2931 | uncharacterized protein LOC118276751 isoform X1 | 0 | 98.76 |
| SNP_2936 | uncharacterized protein LOC118276986 | 0 | 94.16 |
| SNP_2968 | transient receptor potential channel pyrexia-like | 0 | 94.82 |
| SNP_2971** | KH domain-containing, RNA-binding, signal transduction-associated protein 2-like isoform X1 | 0 | 98.67 |
| SNP_2982 | serine protease Hayan-like isoform X2 | 0 | 90.56 |
| SNP_3006 | far upstream element-binding protein 1-like isoform X2 | 0 | 97.96 |
| SNP_3014* | regulator of G-protein signaling loco-like | 0 | 99.53 |
| SNP_3019* | inverted formin-2-like isoform X1 | 0 | 98.23 |
| SNP_3055 | UPF0518 protein GI14169-like isoform X2 | 0 | 94.9 |
| SNP_3060 | tight junction protein ZO-1-like isoform X3 | 0 | 99.58 |
| SNP_3066 | trithorax group protein osa-like isoform X2 | 0 | 100 |
| SNP_3068* | katanin p60 ATPase-containing subunit A-like 1 isoform X1 | 0 | 98.63 |
| SNP_3091* | microtubule-associated protein Jupiter-like isoform X2 | 1.7E-158 | 95.72 |
| SNP_3120* | rho guanine nucleotide exchange factor 17-like isoform X2 | 0 | 98.94 |
| SNP_3147 | BRCA1-associated RING domain protein 1-like | 0 | 98.96 |
| SNP_3171* | hypothetical protein SFRUCORN_004731 | 0 | 99.78 |
| SNP_3179* | transcriptional activator Myb-like isoform X6 | 0 | 99.18 |
| SNP_3210 | zinc finger protein 525-like | 0 | 98.97 |
| SNP_3213 | uncharacterized protein LOC118280315 | 0 | 95 |
| SNP_3216 | TBC1 domain family member 1 isoform X2 | 0 | 97.82 |
| SNP_3218 | P protein-like isoform X1 | 0 | 96.87 |
| SNP_3230 | ankyrin repeat domain-containing protein 50 isoform X2 | 0 | 95.76 |
| SNP_3241 | plasma membrane calcium-transporting ATPase 2 isoform X1 | 0 | 98.71 |
| SNP_3246 | uncharacterized protein LOC118280573 isoform X2 | 0 | 97.14 |
| SNP_3298 | uncharacterized threonine-rich GPI-anchored glycoprotein PJ4664.02-like | 0 | 100 |
