# Supplementary material for: Population genomics of fall armyworm by genotyping-by-sequencing: Implications for pest management
Source: PLoS One. 2023 Apr 18;18(4):e0284587. doi: 10.1371/journal.pone.0284587 (PMC10112782; doi:10.1371/journal.pone.0284587)
Supplement: S4 Table — Darker orange indicates longer distances. (DOCX) [file pone.0284587.s004.docx]

# **S4 Table. Geographic distance matrix showing the straight-line distances (Km) between locations. Darker orange indicates longer distances.**

|  | BA02 | BA03 | DF | GO | MT01 | MT02 | SP | PR | AR01 | AR02 | AR03 | MA01 | MA02 | RS |
| --- | --- | --- | --- | --- | --- | --- | --- | --- | --- | --- | --- | --- | --- | --- |
| BA03 | 75 |  |  |  |  |  |  |  |  |  |  |  |  |  |
| DF | 426 | 487 |  |  |  |  |  |  |  |  |  |  |  |  |
| GO | 827 | 872 | 440 |  |  |  |  |  |  |  |  |  |  |  |
| MT01 | 1347 | 1345 | 1157 | 854 |  |  |  |  |  |  |  |  |  |  |
| MT02 | 1327 | 1328 | 1127 | 816 | 42 |  |  |  |  |  |  |  |  |  |
| SP | 1291 | 1358 | 874 | 671 | 1408 | 1366 |  |  |  |  |  |  |  |  |
| PR | 1606 | 1662 | 1182 | 820 | 1270 | 1230 | 477 |  |  |  |  |  |  |  |
| AR01 | 2492 | 2527 | 2115 | 1677 | 1475 | 1457 | 1607 | 1133 |  |  |  |  |  |  |
| AR02 | 3098 | 3153 | 2674 | 2297 | 2444 | 2415 | 1875 | 1492 | 1127 |  |  |  |  |  |
| AR03 | 2553 | 2605 | 2132 | 1741 | 1931 | 1858 | 1381 | 955 | 728 | 568 |  |  |  |  |
| MA01 | 1060 | 990 | 1470 | 1796 | 1972 | 1934 | 2345 | 2614 | 3335 | 4090 | 3529 |  |  |  |
| MA02 | 1108 | 1038 | 1518 | 1844 | 1889 | 1976 | 2393 | 2662 | 3380 | 4138 | 3577 | 1.4 |  |  |
| RS | 2213 | 2272 | 1787 | 1444 | 1800 | 1763 | 966 | 625 | 1040 | 910 | 475 | 3235 | 3283 |  |
| SC | 1785 | 1847 | 1360 | 1052 | 1561 | 1521 | 523 | 291 | 1242 | 1353 | 875.3 | 2821 | 2869 | 443 |
