# Supplementary figures and images for: Population genomics of fall armyworm by genotyping-by-sequencing: Implications for pest management
Source: PLoS One. 2023 Apr 18;18(4):e0284587. doi: 10.1371/journal.pone.0284587 (PMC10112782; doi:10.1371/journal.pone.0284587)

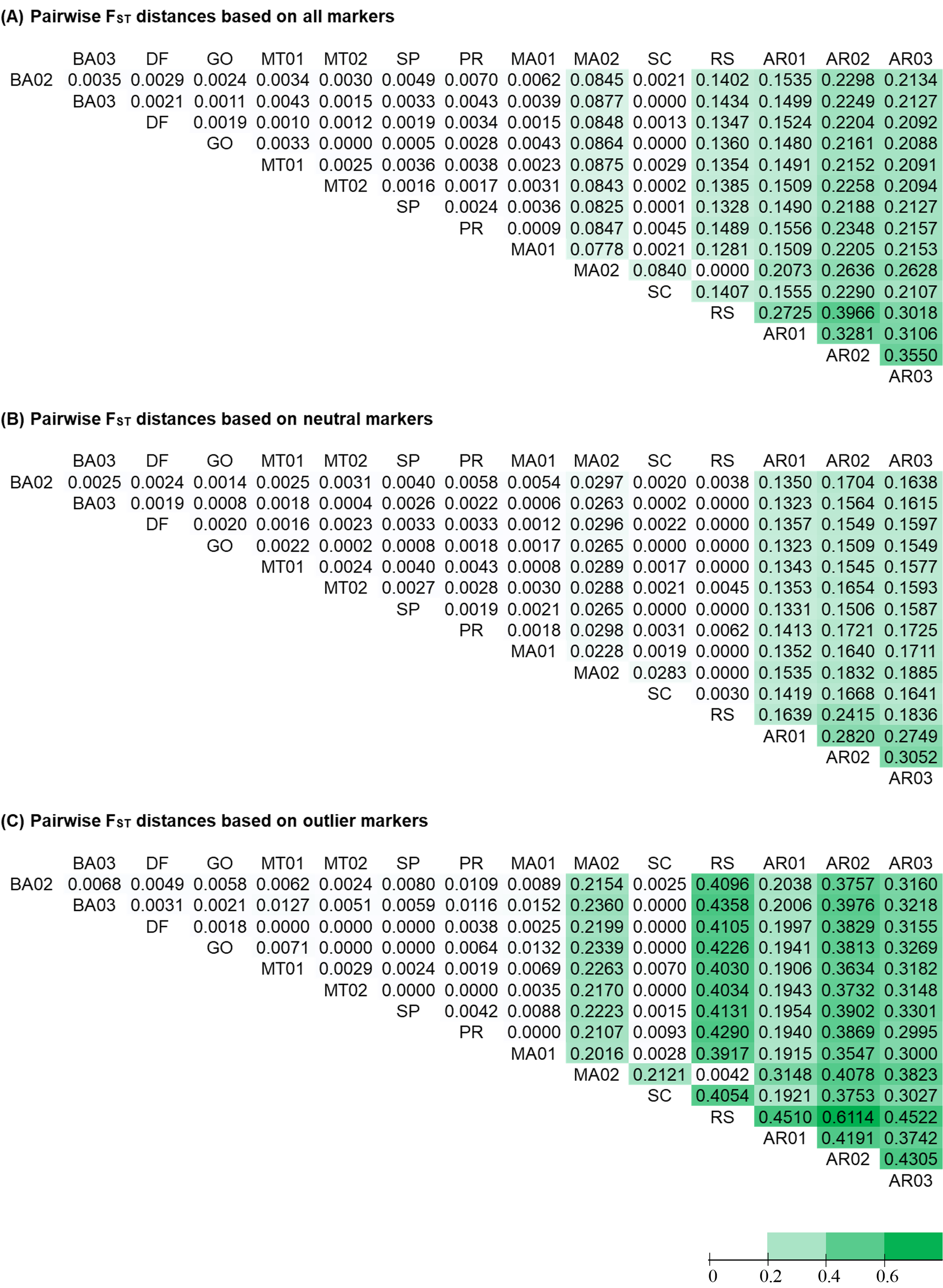

Supplement: S1 Fig — (A) FST calculated with all variant loci (3309 SNPs). (B) FST calculated using neutral markers (2853 SNPs). (C) FST was calculated using candidates putatively under positive selection (456 SNPs) obtained by three methods (FLK, PCAdapt, FstHet). Darker green color represents a higher degree of differentiation. (TIF) [file pone.0284587.s005.tif]

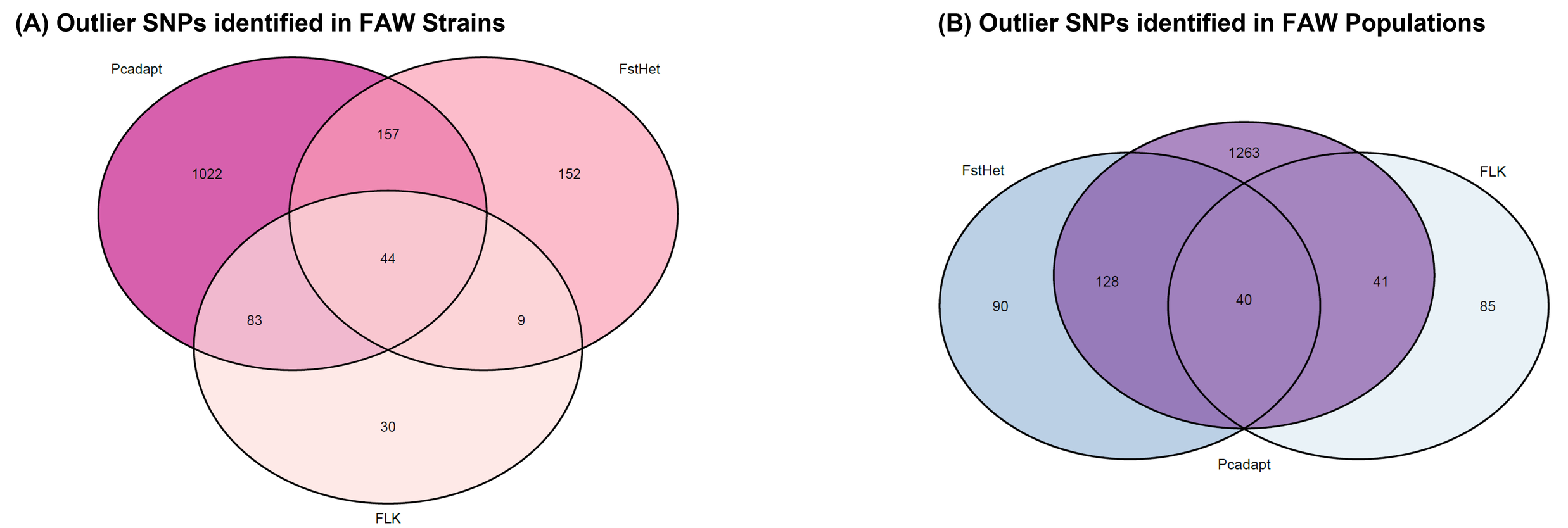

Supplement: S2 Fig — The Venn diagrams shows the number of outlier SNPs associated to (A) FAW host strains and (B) populations. (TIF) [file pone.0284587.s006.tif]

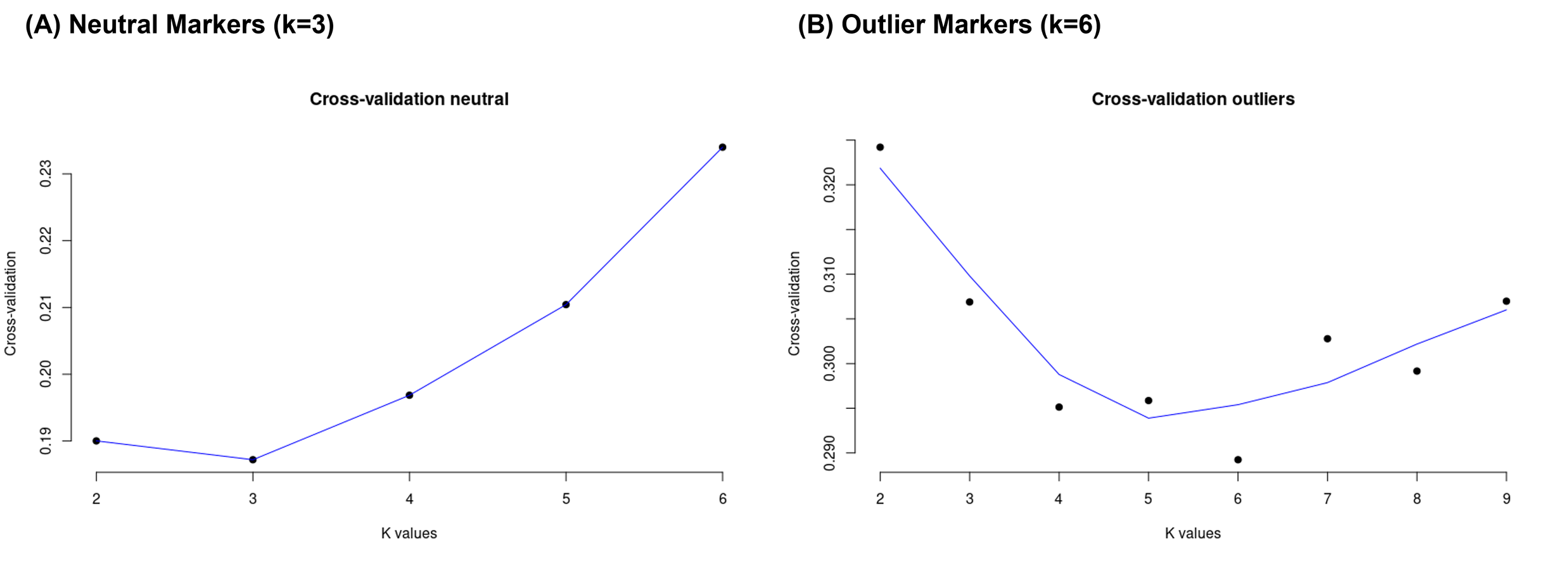

Supplement: S3 Fig — (TIF) [file pone.0284587.s007.tif]

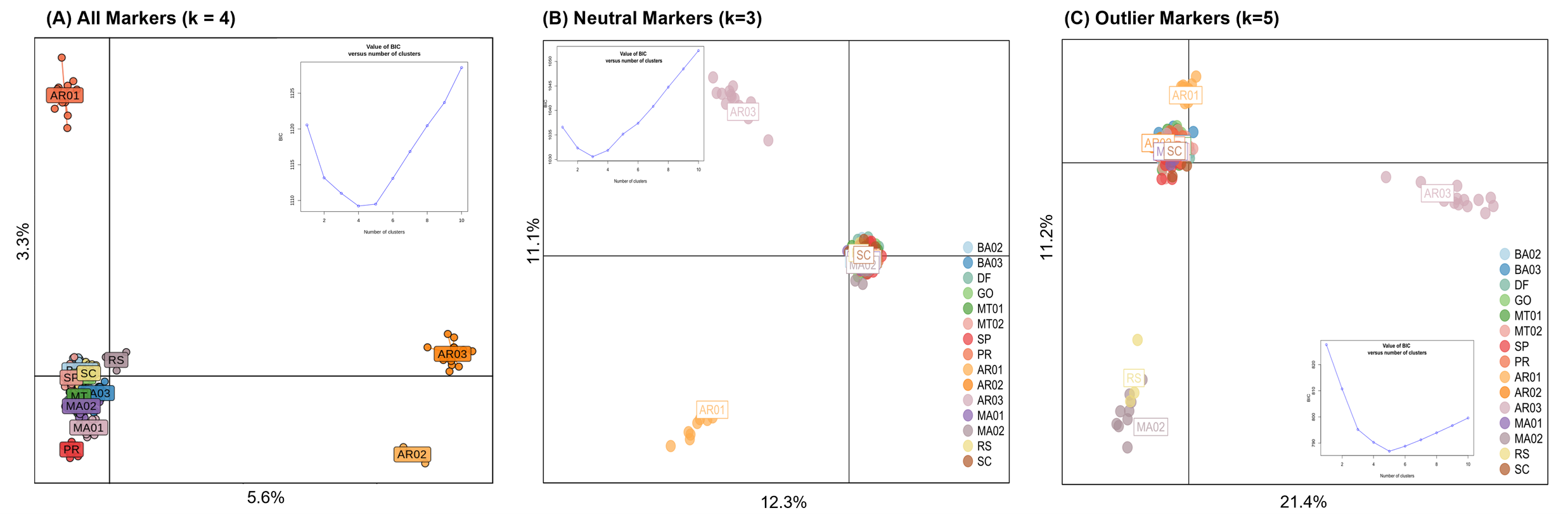

Supplement: S4 Fig — Geographic locations are represented by different colors, and dots represent different individuals. The inset shows BIC values for different number of k clusters. Analysis performed with (A) All SNP loci, (B) 2,853 neutral SNPs, (C) 456 outliers. Plots generated using adegenet package for R software. Sampling locations were considered as priori groupings. (TIF) [file pone.0284587.s008.tif]

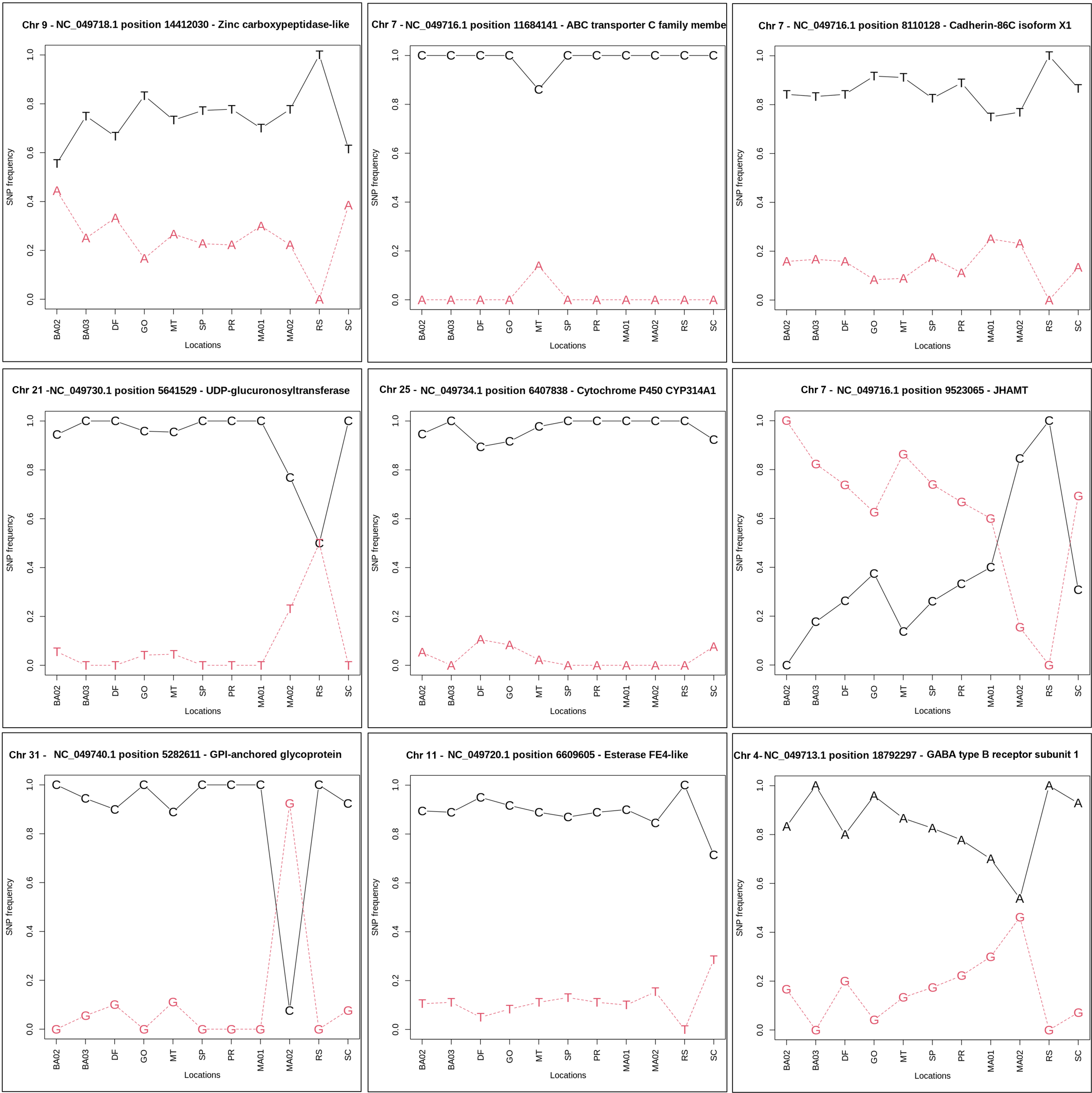

Supplement: S5 Fig — Locations were represented mostly by C-strain moths, except for MA02 and RS locations where most samples were identified as R-strain. (TIF) [file pone.0284587.s009.tif]
